# Genome wide association mapping for agronomic, fruit quality, and root architectural traits in tomato under organic farming conditions

**DOI:** 10.1186/s12870-021-03271-4

**Published:** 2021-10-22

**Authors:** Pasquale Tripodi, Salvador Soler, Gabriele Campanelli, María José Díez, Salvatore Esposito, Sara Sestili, Maria R. Figàs, Fabrizio Leteo, Cristina Casanova, Cristiano Platani, Elena Soler, Aldo Bertone, Leandro Pereira-Dias, Daniela Palma, Resurrección Burguet, Andrea Pepe, Elena Rosa-Martínez, Jaime Prohens, Teodoro Cardi

**Affiliations:** 1 CREA Research Centre for Vegetable and Ornamental Crops, Via dei Cavalleggeri 25, 84098 Pontecagnano Faiano, SA Italy; 2 Instituto de Conservación y Mejora de la Agrodiversidad Valenciana, Universitat Politècnica de València, 46022 Valencia, Spain; 3 CREA Research Centre for Vegetable and Ornamental Crops, Monsampolo del Tronto, AP Italy

**Keywords:** Tomato, Phenotyping, Genome-wide association mapping, Organic farming, Genotype by environment

## Abstract

**Background:**

Opportunity and challenges of the agriculture scenario of the next decades will face increasing demand for secure food through approaches able to minimize the input to cultivations. Large panels of tomato varieties represent a valuable resource of traits of interest under sustainable cultivation systems and for genome-wide association studies (GWAS). For mapping loci controlling the variation of agronomic, fruit quality, and root architecture traits, we used a heterogeneous set of 244 traditional and improved tomato accessions grown under organic field trials. Here we report comprehensive phenotyping and GWAS using over 37,300 SNPs obtained through double digest restriction-site associated DNA (dd-RADseq).

**Results:**

A wide range of phenotypic diversity was observed in the studied collection, with highly significant differences encountered for most traits. A variable level of heritability was observed with values up to 69% for morphological traits while, among agronomic ones, fruit weight showed values above 80%. Genotype by environment analysis highlighted the strongest genotypic effect for aboveground traits compared to root architecture, suggesting that the hypogeal part of tomato plants has been a minor objective for breeding activities. GWAS was performed by a compressed mixed linear model leading to 59 significantly associated loci, allowing the identification of novel genes related to flower and fruit characteristics. Most genomic associations fell into the region surrounding *SUN, OVATE*, and *MYB* gene families. Six flower and fruit traits were associated with a single member of the SUN family (*SLSUN31*) on chromosome 11, in a region involved in the increase of fruit weight, locules number, and fruit fasciation. Furthermore, additional candidate genes for soluble solids content, fruit colour and shape were found near previously reported chromosomal regions, indicating the presence of synergic and multiple linked genes underlying the variation of these traits.

**Conclusions:**

Results of this study give new hints on the genetic basis of traits in underexplored germplasm grown under organic conditions, providing a framework for the development of markers linked to candidate genes of interest to be used in genomics-assisted breeding in tomato, in particular under low-input and organic cultivation conditions.

**Supplementary Information:**

The online version contains supplementary material available at 10.1186/s12870-021-03271-4.

## Background

The expectations of the society for the next decades on the agricultural systems rely on food chains able to guarantee healthy and secure food productions. While the needs concern the adequate levels of supply for the expected growing population, food availability and security are threatened by climate changes and increasing pressures on natural resources [1]. The awareness of more sustainable food systems towards the reduction of the loss of biodiversity and environmental degradation increased the attention to organic farming as a system able to minimize excess of external chemical inputs such as synthetic fertilizers and pesticides [2]. The organic agriculture sector is developing rapidly and its acreage increased by 55% over the past decade, covering globally over 1.5% of the total farmland. In this frame, the European Union represents the second biggest market with Spain, France, and Italy ranking among the eight World top countries in terms of organic cultivation acreage [3].

Tomato (*Solanum lycopersicum* L.) is one of the most important horticultural crops largely appreciated for its high health and nutritional contribution and for its economic value. As a vegetable crop, it represents one of the pillars of the Mediterranean diet being second only to potato in terms of total production [4]. Since the beginning of the twentieth century, intense breeding activities have been principally focused on the development of morphologically diverse and high-yielding varieties [5]. These efforts have been mostly addressed for high input conditions while few attempts have been carried out for low input and/or organic farming. It has been estimated that over 95% of organic production is based on varieties bred for the conventional sector and lacking relevant features for the organic low-input production condition [6]. Therefore, there is an urgent need to establish breeding programs for the development of resilient cultivars able to increase the competitiveness of organic systems [6]. A large reservoir of traits is present in the existing array of traditional varieties adapted to natural and/or marginal environments in which they have been differentially selected by local farmers across years. This germplasm is considered highly valuable in terms of characters related to agro-ecological adaptation as well as consumer preferences [7]. Indeed, the related phenotypes are the complex sum of factors somehow connected to the evolutionary and developmental pressure that determined the adaptability in response to diverse environmental conditions [8]. A comprehensive description of phenotypic profiles is the prior step for promoting the use of the extant diversity as well as a cornerstone for establishing new breeding programs. Therefore, its exploration is fundamental for adding value to local genotypes rediscovering those traits lost in modern varieties that have been mostly developed for their yielding capacity and attitudes to processing [9].

The increasing availability of large-scale genotypic data of germplasm resources for many crops has been benefited by the innovation in genomics and cutting-edge genotyping platforms. Linking phenomic information to genomic sequence data has provided invaluable opportunities for the dissection of the genetic basis of complex traits implemented in genome-wide association mapping studies (GWAS). GWAS has emerged as a powerful approach for the identification of genetic variants present at a significantly higher frequency for a target phenotype in unrelated individuals [10]. The advantage over bi-parental mapping population relies on a broader genetic base to exploit and the higher map resolution due to the larger number of recombination events occurring in natural populations. Since its development, the GWA computational efficiency has been bolstered by integrating the population structure covariates and the family kinship inference into the linear mixed model (LMM). This approach has been successfully applied in crops for both morphologic and agronomic traits [11]. It reduces the rate of false positives increasing the power of association through correction based on the relationships of individuals [12]. Furthermore, the compression approach (CLMM) and the population parameters previously determined (P3D), by clustering individuals into groups and eliminating the re-computation of variance components, improves statistical power by reducing bias due to any occurring substructures in genetic association datasets [13].

Tomato has been a pioneering crop for QTL mapping on crossing populations for agronomic and yield related traits. In the last decade, several GWA studies have been performed resulting in the identification of novel loci mostly for fruit quality, metabolites and flavor-related compounds [14–16]. Further efforts to investigate major agronomic traits have been performed in core sets of improved and wild cultivars [17]. More recently, studies have been directed to the dissection of several morpho-agronomic and plant architecture features in panels of approximately 160 wild, cultivated and cerasiforme genotypes [18] as well as in 126 landraces and vintage cultivars [19].

All those studies have been focalized on the epigeous part of the plant leading to the identification of several associated genomic regions, whereas fewer attempts have been done for root traits. The root system is critical for water absorption and nutritional elements supply, providing physical support for plant growth. Most of the improved cultivars being adapted to high input conditions result in the development of smaller root systems [20]. Exploring root architecture, therefore, facilitates the identification of genotypes able to perform more efficiently in suboptimal conditions such as limited availability of nutrients, drought, or salinity.

In the present study, we performed a broad phenotypic characterization for morphological, agronomic, and root traits in 244 cultivated tomato genotypes grown in two organic field trials under typical Mediterranean environmental conditions. Then we linked the diversity observed at the phenotypic level with genomic data obtained through Restriction Site Associated DNA Sequencing by implementing a GWAS analysis. The population’s size used is adequate to provide a comprehensive catalogue of phenotypic information and to improve the detection power of GWAS. The obtained results will shed light on the potentiality of the collection studied for the organic farming sector and for planning further breeding programs. Furthermore, the novel associated regions may provide useful insight into functional studies.

## Results

### Phenotypic diversity

**Fig. 1:**
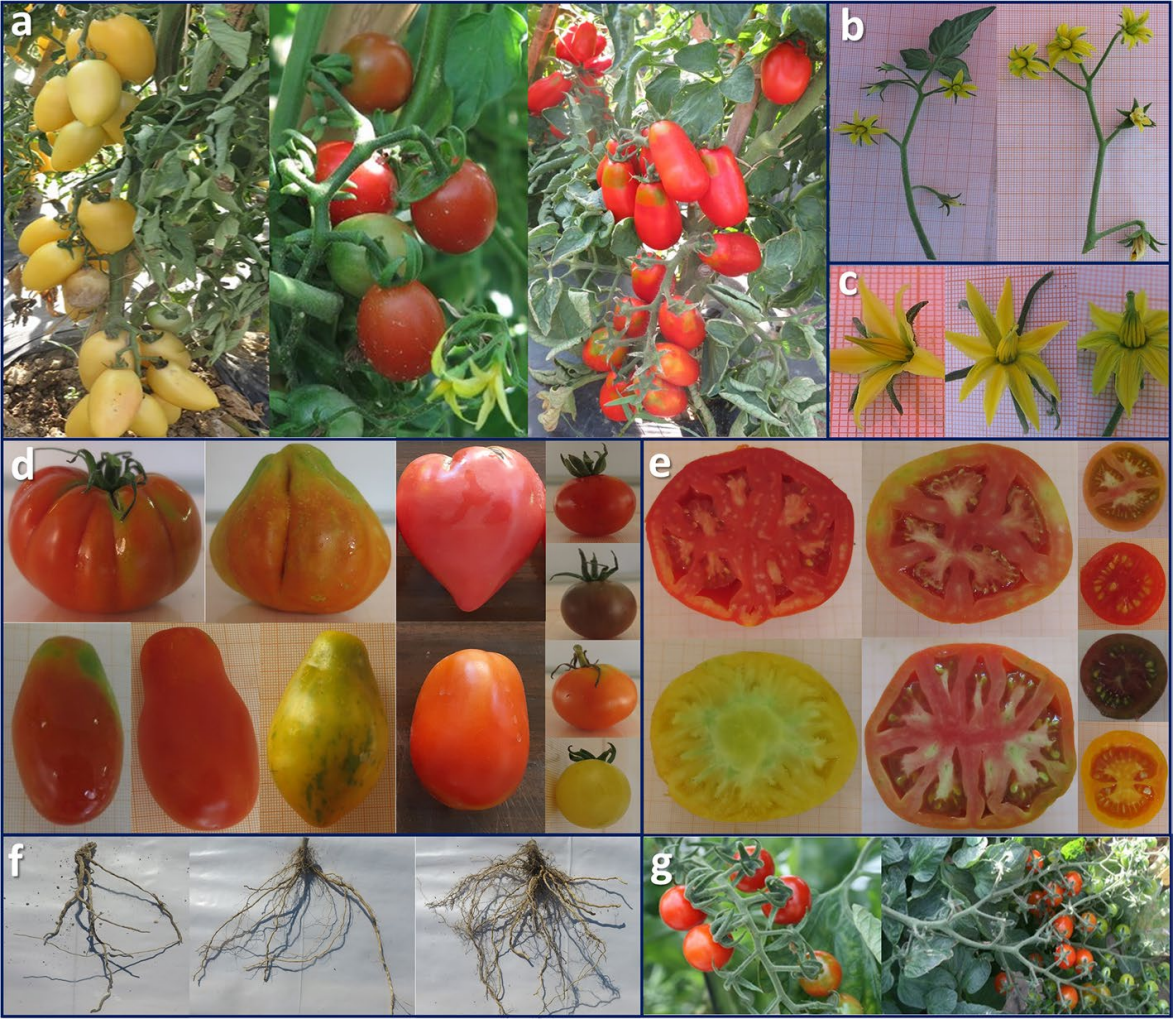
Overview of the phenotypic diversity of the collection studied. **a** Fruits on the plant and their diversity; **b** Leafy and not leafy inflorescence; **c** Flowers with exerted and inserted styles; **d** Variability for fruit size, shape, colour and green shoulder; **e** Puffiness and locules number; **f** Density of fine roots and radicular crown angle; **g** Type of inflorescence with mature fruits: uniparous and compound

A wide range of phenotypic diversity was observed in the considered germplasm collection (Fig. 1). Significant differences between accessions (*P* < 0.001) were found for 25 traits, whereas pests in foliage and fruits showed a lower significance threshold (*P* < 0.01) (Table 1). Only the length and diameter of the three largest roots, as well as the diseases in foliage and fruits, were not significant. On average, higher heritability was found for morphological plant and fruit traits, although two agronomic traits, fruit set/truss and fruit weight displayed heritability values above 70 and 80%, respectively. For all traits, the coefficient of phenotypic variation was from moderate to high with values ranging from 9.56% (pH) to 176.72% (fruit weight). Values of the phenotypic coefficient of variance (PCV) were higher than those of the genotypic coefficient of variance (GCV), with a ratio PCV/GCV ranging from 1.02 to 1.52 for fruit weight and disease in fruits, respectively. The highest GCV values were found for puffiness appearance, fruit set/truss and fruit weight (Table 1). These traits also exhibited the highest genetic advance as a percentage of the mean (GAM) with values above 98%. On average, GAM was comparable between morphological and agronomic traits, although, slightly higher in the former. Lower general values were instead observed for root traits. The lowest levels (< 10%) were observed for pH and Radicular Crown Angle.

**Table 1:** Descriptive statistics for pseudoqualitative and quantitative traits, broad sense heritability (*H*^2^), phenotypic and genotypic coefficients of variation (PCV and GCV, respectively), and genetic advance as percent of population mean (GAM), for traits analysed in two environments (Alcasser, Spain; Monsampolo del Tronto, Italy) on 244 tomato cultivars

Trait	Acronym	Rsquare	Fratio	***Prob*** > F^**a**^	***H***^***2***^	PCV	GCV	GAM
**Morphological plant and fruit descriptors**								
Foliage Density	FD	0.45	4.09	***	44.89	48.77	43.72	24.59
Plant Vigor	PV	0.31	2.29	***	31.36	46.59	38.87	18.28
Leafy Inflorescence	LI	0.69	11.23	***	69.11	102.00	97.74	68.21
Style Position	SP	0.45	4.14	***	45.21	89.31	80.16	45.24
Fruit Set Sequence	FSs	0.24	1.59	***	24.09	53.03	41.57	16.78
Green Shoulder	GS	0.67	10.03	***	66.64	143.52	136.86	93.81
Puffiness Appearance	PA	0.58	7.05	***	58.40	163.81	153.29	98.36
Fruit Firmness	FF	0.38	3.02	***	37.54	47.10	40.82	21.00
**Agronomic traits**								
Ribbing at Calyx End	RC	0.68	10.81	***	68.29	99.75	95.43	66.21
Radial Cracking	RK	0.43	3.81	***	43.17	115.03	102.38	56.48
Concentric Cracking	CC	0.38	3.12	***	38.30	146.12	127.14	66.06
Fruit fasciation	FFA	0.39	3.25	***	39.27	134.91	117.96	62.07
Blossom-end Rot	BR	0.22	1.41	***	21.94	100.86	77.16	30.35
Pests in Foliage	PF	0.21	1.30	**	20.61	88.76	66.77	25.45
Pests in Fruits	PFr	0.21	1.31	**	20.74	111.32	83.88	32.07
Disease in Foliage	DF	0.18	1.11	*NS*	18.05	97.85	70.91	25.29
Disease in Fruits	DFr	0.13	0.76	*NS*	13.11	104.98	68.92	20.95
Locules number	LN	0.69	11.01	***	68.68	124.40	119.11	82.88
Ripening Earliness	RE	0.58	6.95	***	58.07	30.68	28.68	18.35
Ripening Uniformity	RU	0.22	1.42	***	22.02	114.37	87.58	34.50
Fruit Weight	FW	0.81	21.47	***	81.05	176.72	172.74	130.54
Total Yield	TY	0.46	4.28	***	46.03	103.60	93.28	53.10
Fruits Set/ Truss	FST	0.71	12.23	***	70.89	166.40	159.99	110.73
Soluble solids content	SS	0.53	5.61	***	52.77	39.04	35.97	21.94
pH	PH	0.42	3.58	***	41.61	9.56	8.45	4.57
Acidity	AC	0.33	2.51	***	33.36	52.38	44.31	21.44
**Root characterization**								
Radicular Crown Angle	RA	0.23	1.50	***	23.02	29.66	22.98	9.26
Length of 3 Largest Roots	RL	0.14	0.79	*NS*	13.58	49.89	33.13	10.25
Diameter of 3 Largest Roots	RD	0.19	1.16	*NS*	18.83	60.00	44.01	16.03
Diameter of Main Root	DMR	0.31	2.30	***	31.46	52.43	43.78	20.61
Density of Fine Roots	DR	0.33	2.48	***	33.03	68.00	57.39	27.69

^a^ ***, ** indicate significance at *P* < 0.001, *P* < 0.01, respectively; *NS* not significant

### Morphological plant and fruit descriptors

Regarding traits in qualitative scale, the collection was largely composed of indeterminate accessions with inflorescences mostly uniparous or compound with a reduced number of accessions having a fishbone inflorescence (Additional File 1: Figure S1). The external fruit colour at maturity was mostly red with several long shelf life (LS) accessions having pink external colour. Genotypes with yellow fruits were instead found among heirlooms, landraces for fresh consumption and cultivars. A wide diversity was found for berry morphology with a different rate of variability between accessions groups (AG) being higher in heirlooms (HL) and lower in breeding lines (BL). ​We observed the occurrence of all the classes of shape, although the most represented were the flattened or circular ones. Blossom end scar was mostly closed in all AG with a high proportion of cultivars, landraces for fresh consumption as well as heirlooms holding both open and closed blossom end. (Additional File 1: Figure S1).

**Fig. 2:**
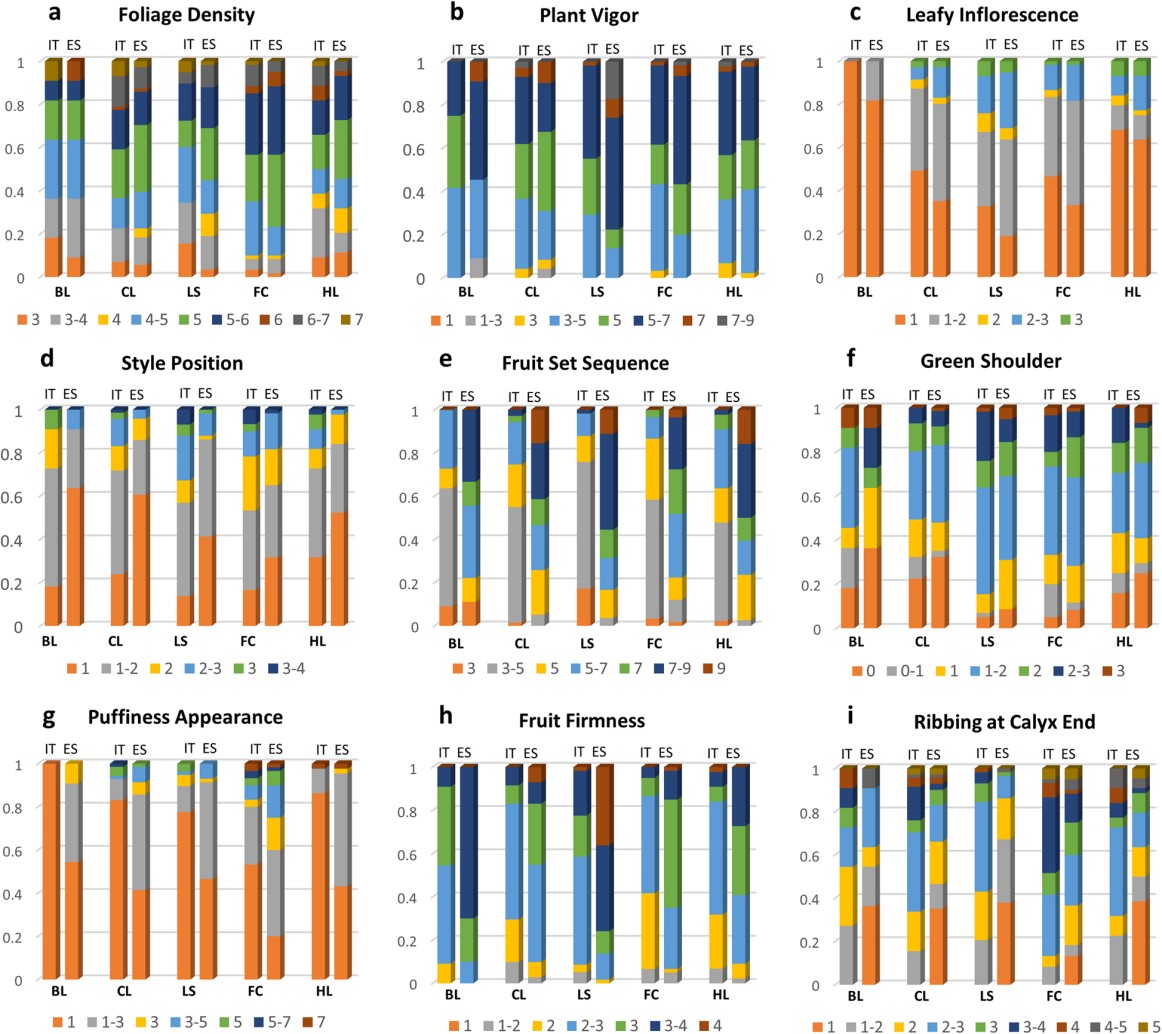
Variation for morphological plant and fruit descriptors across cultivar groups in two locations. Stacked bars indicate the proportion of each class for the considered traits on a total scale 0–1. **a-d** Plant and flower traits; **e-i** Fruit traits. Details of measurement scale for each trait are in Supplementary Table 2. Locations: IT = Italy, ES = Spain; Cultivar groups: BL = breeding lines, CL = elite cultivars, LS = long shelf-life landraces, FC = landraces for fresh consumption, HL = heirloom varieties

As for pseudo-qualitative traits, many accessions showed intermediate foliage density and a medium or strong vigor of plants (Fig. 2a, b). Few accessions had leafy inflorescences with shoots, whereas the style position was mostly inserted or at the same level as the stamens favoring self-pollination (Fig. 2c, d). The fruit set sequence was on average higher in Spain for all accession groups (Fig. 2e), whereas less differences were found for green shoulder which ranged from absent to dark green, with most accessions having light green or medium green shoulders (Fig. 2f). Remarkably, the accessions had a low level of puffiness and medium level of firmness (Fig. 2g, h). Larger diversity was instead observed for the ribbing at the calyx end, although only a minor portion of genotypes had a strong or very strong ribbing (Fig. 2i).

For three morphological traits (style position, external colour, plant vigor), the range of variation did not cover the entire descriptors scale, in fact, the evaluated panel did not include individuals with highly exerted style position, green colour of mature fruits, and very strong plant vigor. Among accessions groups, breeding lines did not show any accessions with shoots in leafy inflorescence, very good fruit set sequence, and severe puffiness, while landraces for fresh consumption and heirlooms encompassed the largest variability in fruit shapes (Additional File 1: Figure S1, Table S1).

### Agronomic traits

**Fig. 3:**
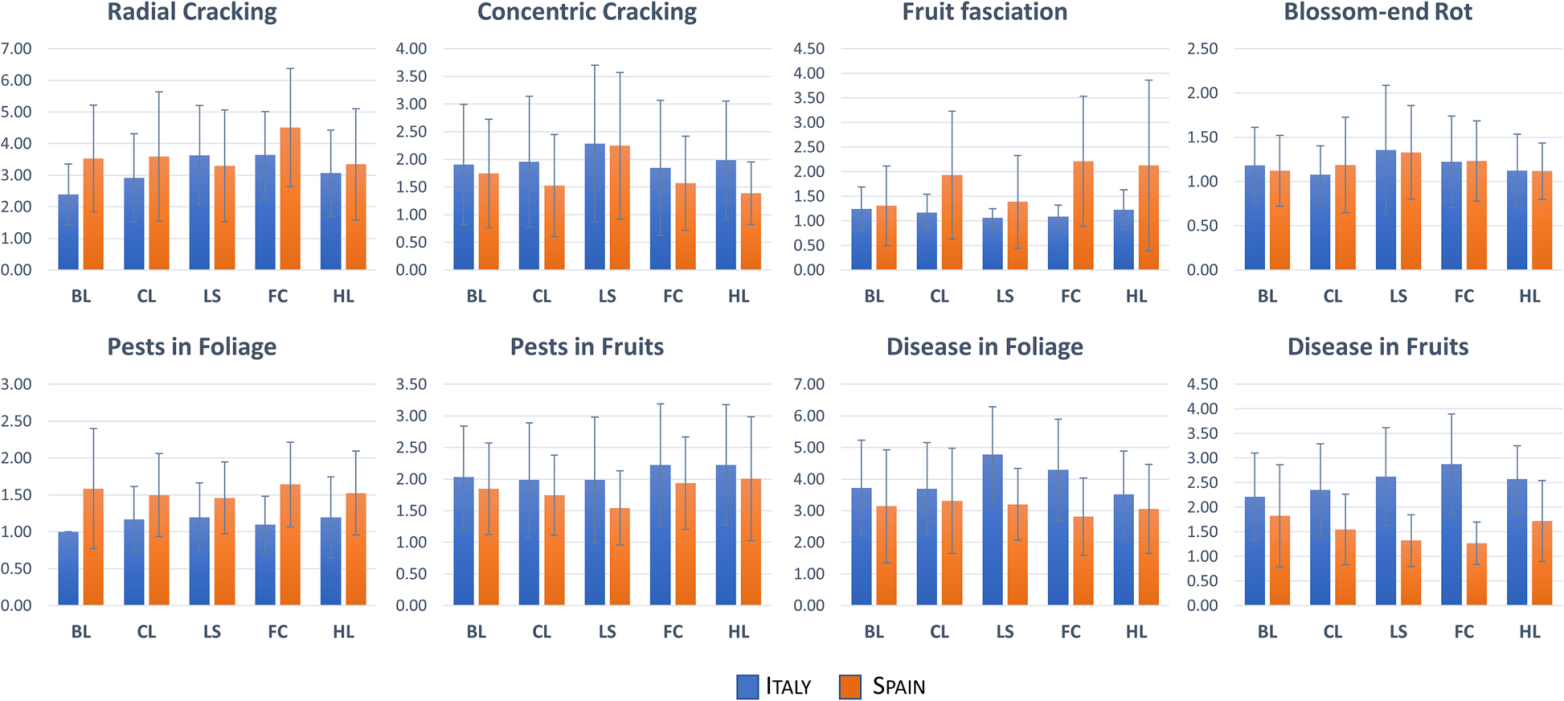
Assessment of cultivar groups in Italy and Spain for (pseudo) qualitative agronomic traits. Histograms with error bars indicating ± standard deviations, showing average values for each cultivar group in each location. Qualitative measures are reported on the Y-axis for each trait. For all traits related to agronomic fruit quality, the scale varies from 1 (absent) to 7 (abundant); for pests and diseases in foliage, the scale varies from 1 (very scarce) to 9 (very severe). For cultivar group codes, see Fig. 2

Plants and fruits were evaluated for agronomic traits, including their global health status during trials conduction. All accessions showed a slightly higher level of radial cracking with respect to concentric cracking (Fig. 3) with similar levels at both locations. The same trend was observed for blossom-end rot which was generally absent or low at both locations. On the contrary, fruit fasciation resulted higher in Spain reaching intermediate or severe values in several accessions of the considered cultivar classes. At both sites, the lowest levels of fruit fasciation were found for long shelf-life types, with no significant differences among cultivar groups in Italy (Table S1). The incidence of symptoms of pests in foliage was very low in all cultivar groups with higher values in Spain than in Italy. The opposite trend was observed for pests in fruits and diseases in foliage and fruits, all showing higher average values in Italy. Overall, the incidence of pests and diseases was low and quite similar among varietal groups, with no significant differences observed for pests in Italy and for disease in foliage in Spain.

**Fig. 4:**
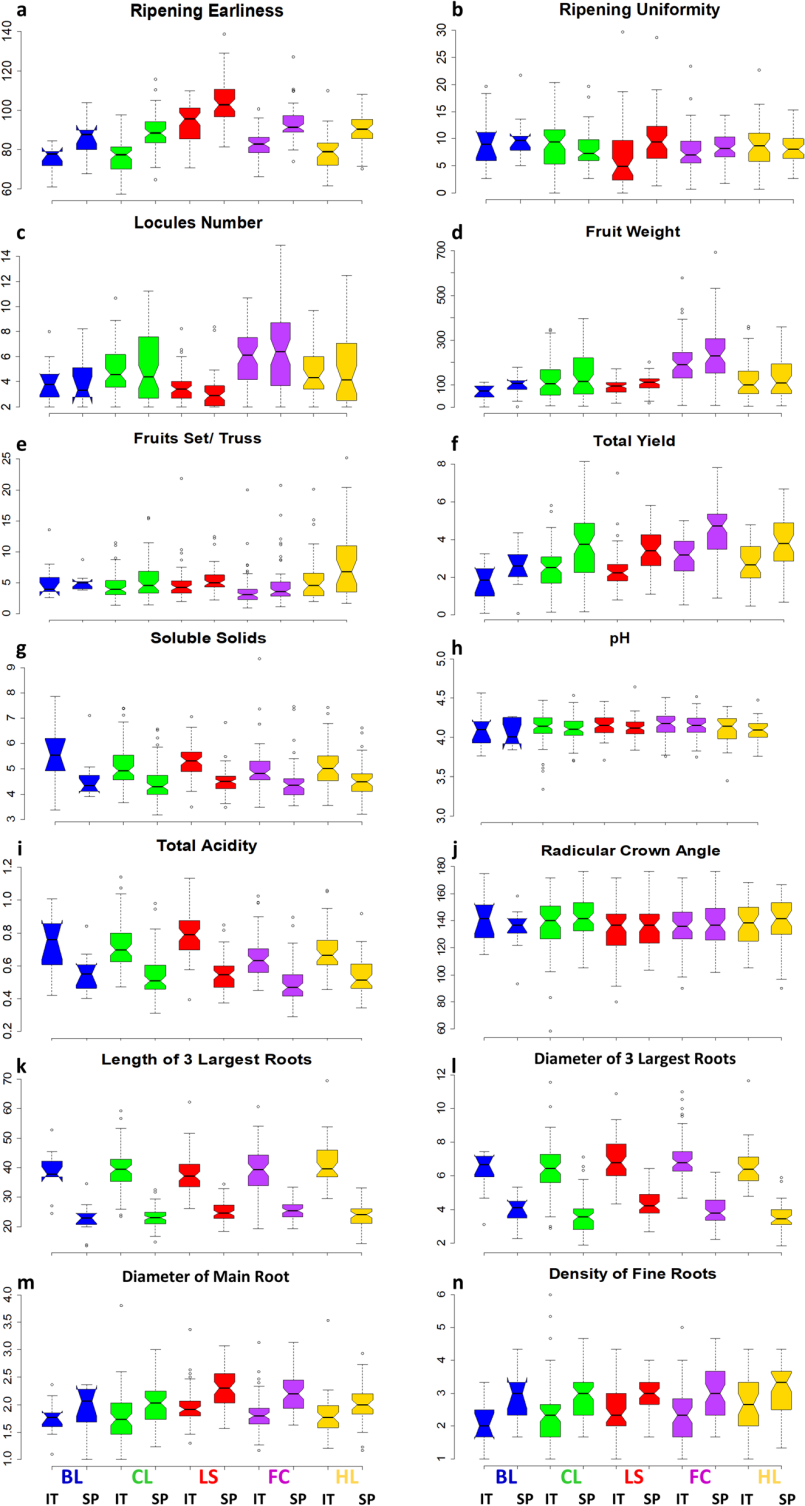
Quantitative agronomic and root trait characterization. Notched box-plots showing median values and quartiles for the different cultivar group in each location. The measurement scale for each trait is reported on the Y-axis, details in Supplementary Table 2. **a-b** ripening related traits; **c-f** yield related traits; **g-i** chemical traits; **j-n** root traits

Marked divergences were found among accessions groups and locations for quantitative trait performances (Fig. 4). An earlier fruit maturation was observed in Italy with an average difference of 11 days over Spain (Fig. 4a, Table S2). Among cultivar groups, the LS types matured later than the others at both locations. Ripening uniformity was instead homogeneous for all accessions (Fig. 4b) being 7.99 and 8.72 days in Italy and Spain, respectively (Table S2).

In average, landraces for fresh consumption had the largest locules number as well as the greatest variability (Fig. 4c). The same trend was observed for fruit weight which, in addition, exhibited the highest value in all cultivar groups in Spain (Fig. 4d).

For both fruit weight and number of fruits per truss (Fig. 4e), the variation among accessions was very high, reaching differences of 500-fold between individuals with the largest and the smallest berry. We observed overall yields higher in Spain than in Italy with average increases in all cultivar groups ranging from 39% for the Heirlooms to 47% for LS types (Fig. 4f, Table S1). On the contrary, the soluble solids content was higher in Italy than in Spain with average values of 5.13 and 4.49, respectively (Fig. 4g, Table S2). On average, breeding lines had the highest average values for soluble solids content although landraces for fresh consumption with high values were found at both locations (Table S1). In general, the accessions with high soluble solids content had very low fruit weight (e.g., BT00500 showed the highest soluble solid content with 9.35 of Brix° and fruit weight 8.72 g in Italy and 7.46 of Brix° and fruit weight 9.32 g in Spain; data not shown). Although the pH was almost similar among accessions not showing any significant differences between growth sites, the total acidity was higher in Italy for all accession groups (Fig. 4h, i). Overall, in both environments, we found several local varieties with better performance for yield- and quality-related traits with respect to heirlooms and fresh cultivars.

A remarkable variation was found for root traits. Among them, only radicular crown angle did not show any significant variation between cultivation sites and among accessions groups (Fig. 4j, Table S1). The measures carried out on the 3 main roots exhibited the highest values in Italy for all cultivar groups with average values 61 and 74% higher than in Spain for the length and the diameter, respectively (Fig. 4k, l, Tables S1-S2). On the contrary, the diameter of the main root and the density of fine roots were on average greater in Spain although the highest values were found in some heirlooms and cultivars grown in Italy (Fig. 4m, n, Table S1).

### Genotypic and environmental components underlying trait variation

Results of the combined analysis of variance for the traits evaluated across growing sites are given in Table 2. A high level of significance (*P* < 0.001) was found for the genotypic component (G) for all traits except for pests in fruits and for the field scored diseases. For most traits, the main source of variability was due to G, which accounted on average for 40.46% of the total sums of squares (TSS%), ranging from 13.11% for DFr to 81.05% for FW. Among morphological characteristics, on average G accounted for 47.15%, while for the agronomic and root traits, on average G accounted for 42.06 and 43.75%, of TSS% respectively.

**Table 2:** Analysis of variance and significant levels for genotypic (G) and environmental effects due to location (E) for tested lines and combined effects for the pseudoqualitative and quantitative traits evaluated

Trait acronym	Genotype (G) *df = 243*		Locality (E) *df = 1*		G × E *df = 243*		Error *df = 976*
	TSS%	F	TSS%	F	TSS%	F	TSS%
**Morphological plant and fruit descriptors**							
FD	44.90	4.59***	0.00	0.00^NS^	15.91	1.63***	39.20
PV	31.36	2.78***	1.64	35.42***	21.77	1.93***	45.23
LI	69.07	10.23***	0.39	13.88***	3.35	0.50^NS^	27.20
SP	45.19	5.19***	4.90	136.77***	14.85	1.71***	35.05
FSs	24.09	4.17***	36.10	1518.59***	16.60	2.87***	23.21
GS	66.64	11.16***	0.16	6.35*	9.23	1.55***	23.98
PA	58.40	8.24***	2.30	78.79***	10.86	1.53***	28.45
FF	37.56	4.55***	11.61	342.05***	17.75	2.15***	33.09
**Agronomic traits**							
RC	68.28	16.61***	7.25	428.77***	7.94	1.93***	16.52
RK	43.17	4.32***	0.95	23.16***	15.78	1.58***	40.10
CC	38.30	3.32***	1.05	22.22***	14.36	1.25*	46.28
FFA	39.27	8.24***	10.28	524.25***	31.30	6.57***	19.14
BR	21.93	1.42***	0.02	0.39^NS^	16.19	1.05^NS^	61.86
PF	20.61	1.51***	5.48	97.51***	19.07	1.40***	54.84
PFr	20.74	1.28**	1.01	15.10***	13.04	0.80^NS^	65.21
DF	18.05	1.23*	3.89	64.10***	18.88	1.28**	59.19
DFr	13.11	0.87^NS^	12.45	201.81***	14.23	0.95^NS^	60.21
LN	68.68	12.46***	0.13	5.70*	9.05	1.64***	22.14
RE	57.66	13.47***	16.92	960.29***	7.53	1.76***	17.89
RU	22.02	1.62***	0.33	5.85*	23.12	1.70***	54.54
FW	81.05	23.88***	1.08	77.20***	4.24	1.25*	13.63
TY	46.03	5.62***	10.54	312.72***	10.55	1.29**	32.88
FST	70.89	15.59***	2.97	158.92***	7.87	1.73***	18.26
SS	52.79	7.69***	10.91	386.10***	8.77	1.28**	27.54
PH	41.27	3.73***	0.22	4.84*	13.30	1.21*	45.21
AC	33.39	4.55***	22.26	740.28***	14.74	2.04***	29.61
**Root characterization**							
RA	23.02	1.61***	0.16	2.76^NS^	19.39	1.36***	57.43
RL	13.58	1.62***	41.36	1198.90***	11.39	1.36***	33.67
RD	18.83	2.74***	42.82	1517.60***	10.83	1.58***	27.53
DMR	31.48	2.61***	4.66	93.90***	15.47	1.28**	48.39
DR	33.03	3.14***	6.42	148.17***	18.26	1.73***	42.29

*, **, ***, significant at *P* < 0.05, *P* < 0.01, *P* < 0.001, respectively; *NS* not significant

*df* degrees of freedom, *TSS* total sum of squares, *F* F ratio

Partitioning of TSS% in the other components affecting the variation indicated that E and G × E accounted for an average of 12.61 and 23.99%, respectively, resulting in a generally lower influence on the analyzed parameters. Foliage density, blossom-end rot and radicular crown angle did not show any significant variation due to the site, whereas significance at *P* < 0.05 was found for GS, LN, RU and PH. Four traits including LI, BR, PFr and DFr were not influenced by G × E factors, while a low significance was observed on CC, FW and PH.

The length and the diameter of roots were the most affected by site environmental differences showing a TSS of 41.36 and 42.82%, respectively. The G × E interaction was on average higher for root traits (TSS equal to 15.07%). respect to agronomic (TSS equal to 13.88%) and morphological (TSS equal to 13.79%) ones. Fruit fasciation and ripening uniformity were those for which the variation was highly influenced by G × E with TSS values of 31.30 and 23.12%, respectively.

### Multivariate analysis and correlations

**Fig. 5:**
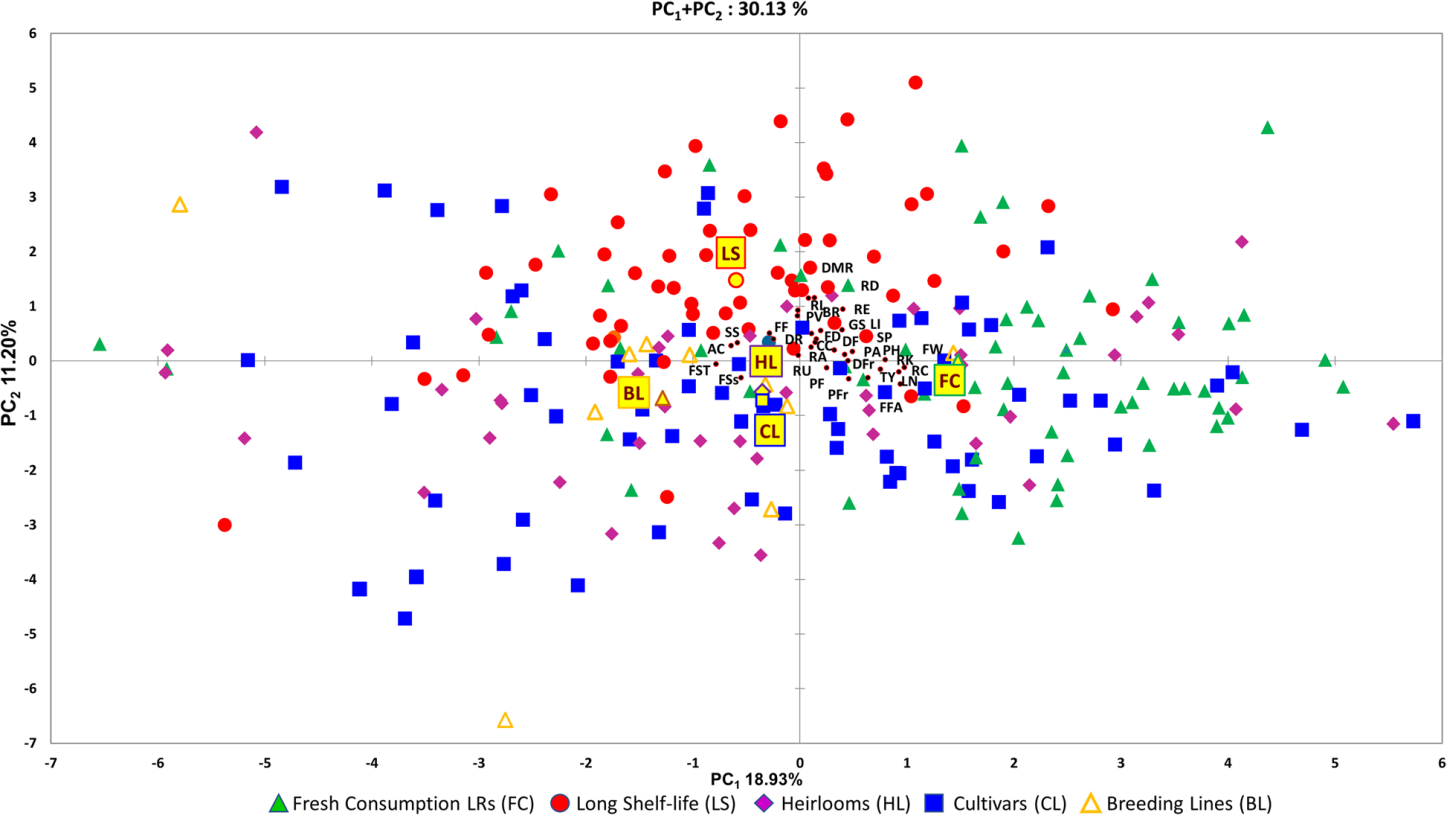
Phenotypic variability of the 244 cultivated tomato genotypes. Scatter plot of the first (PC_1_) and second (PC_2_) principal components showing the variation for 31 pseudo-qualitative and quantitative morphological, agronomic and root traits scored in two environments. Based on cultivar groups, accessions are represented by different coloured symbols indicated in the legend. The first and second component centroids for each cultivar groups are indicated by filled yellow symbols with shape and edge colour according to cultivar groups (see legend). The direction from the centre of the biplot indicate how each trait contributes to the first two components. Trait acronyms are listed in Table 1

The principal component analysis (PCA) in the first two dimensions explained 30.13% of the total variance (Fig. 5, Additional file 1: Figure S2). The first six components explained over 50% of the variation. The first component, accounting for 18.93% of the total variance, was positively correlated to 22 of the traits scored and included all pseudo-qualitative agronomic traits. The second component which explained 11.20% of the total variance was positively correlated to 24 traits and included all root characterizations. Morphological traits and agronomic quantitative measures showed a wide distribution being present in both negative and positive parts of the biplot. Only fruit set sequence and fruit set truss were negatively correlated to both components. The eigenvalues and the variable contribution for principal components are reported in Table S3. Fruit weight and diameter of 3 Largest Roots were the main factors discriminating the genotypes under study accounting for 12 and 17.13% of the total variation of the first and second components, respectively.

The projection of the accessions on the two-dimensional PCA graph evidenced the wide variability of accessions groups according to phenotypic characterizations highlighting a partial differentiation of the long shelf-life types from the rest, which were mostly distributed in the positive axis of the second component. Fresh consumption landraces were instead mainly distributed in the positive loadings of the first component. Cultivars and heirlooms were more interspersed although centroids cluster closely in the negative plot of the first two PCs.

**Fig. 6:**
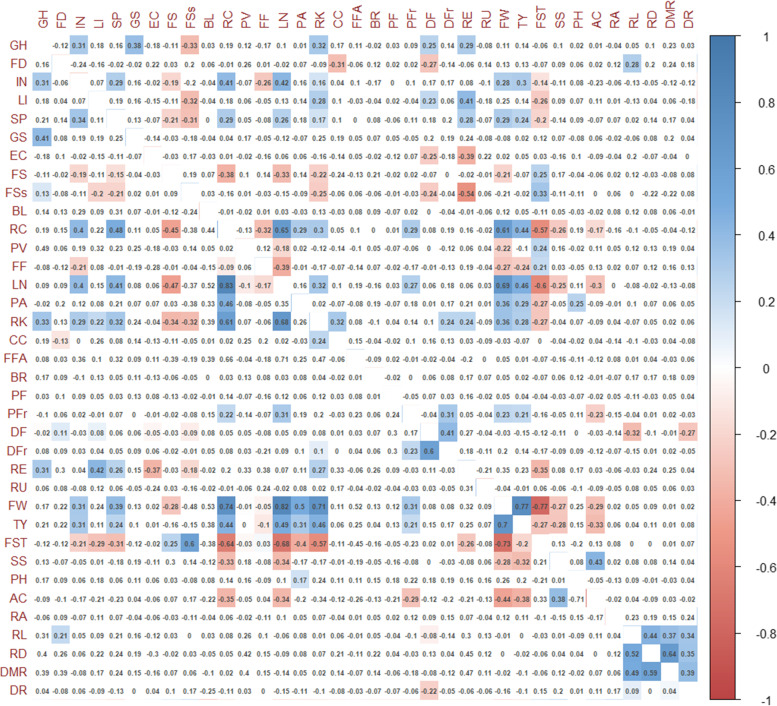
Spearman’s rank correlation coefficients between pairs of phenotypes. Correlation coefficients are indicated in each cell. Coloured correlations are those with *P* value < 0.05 after Bonferroni correction. Colour intensity is directly proportional to the coefficients. On the right side of the correlogram, the legend colour shows the correlation coefficients and the corresponding colours. Correlogram for traits scored in Spain is placed below the diagonal, the correlogram for traits scored in Italy is placed upside the diagonal. Trait acronyms are listed in Table 1

The Spearman rank correlation coefficients after Bonferroni correction calculated for qualitative and quantitative traits revealed how some traits were rather independent, whereas a group of traits clustered together because of a reciprocal tight correlation for the same category of measures (e.g., root traits, yield-related traits) (Fig. 6). Interestingly, we found at both locations positive significant correlations between fruit weight and total yield with different morphological features, suggesting how plant architecture could be a good indicator for the selection of performing genotypes. Soluble solid content was negatively correlated at both sites with yield, fruit weight and locule number. As expected, the number of fruit locules was strongly correlated with the increase of fruit weight. Finally, fruit acidity was negatively correlated with total yield and fruit weight, and positively correlated with soluble solids.

### Population stratification

**Fig. 7:**
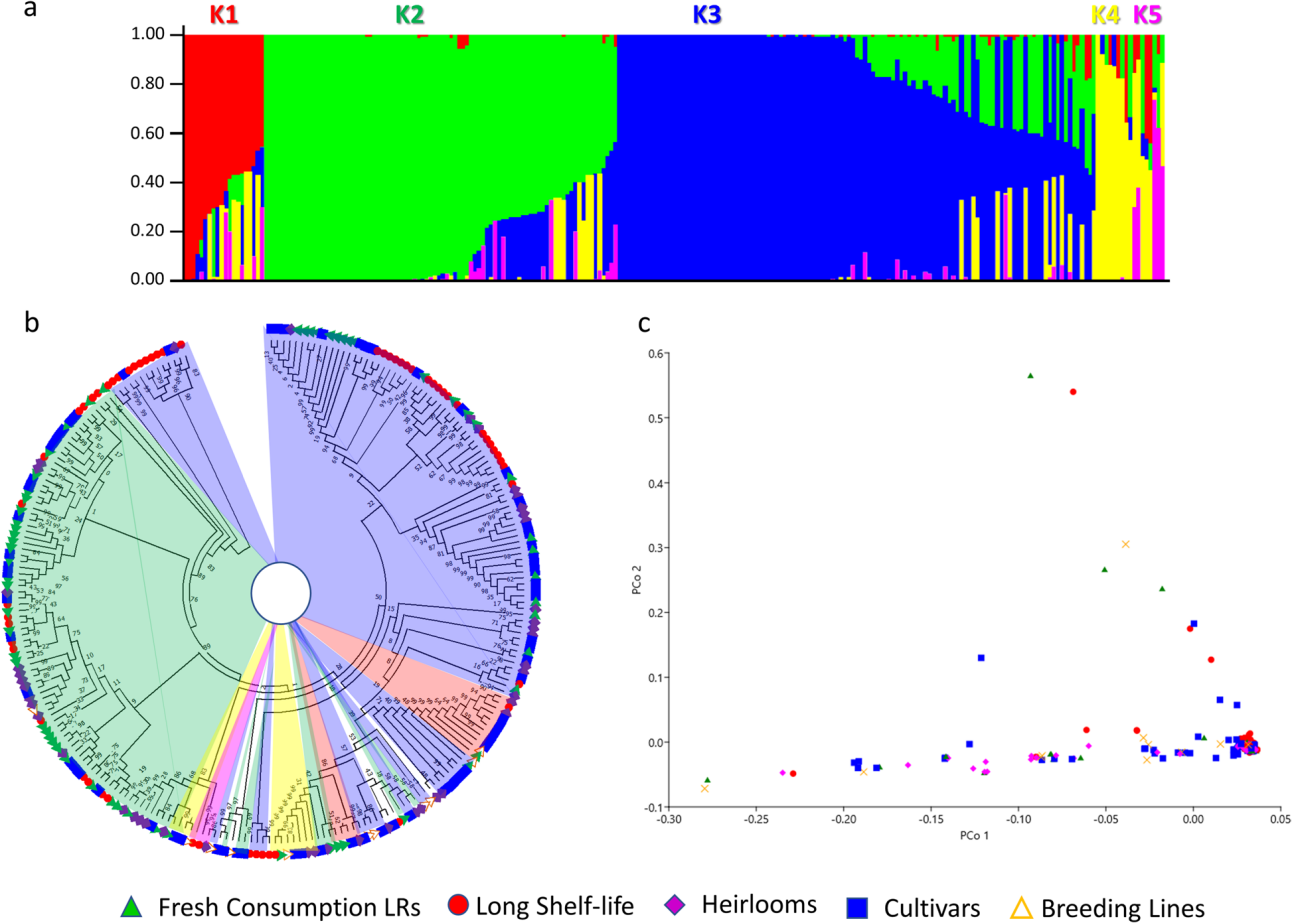
Genomic diversity of the cultivated tomato collection. **a** STRUCTURE analysis of 244 *S. lycopersicum* genotypes with 37,317 SNP markers in the case of five clusters (K). The vertical coordinates of each subpopulation indicate the membership coefficient for each individual; each vertical bar represents one genotype. The coloured blocks correspond to the different clusters. **b** Maximum likelihood phylogenetic tree (unrooted). The initial tree for the heuristic search was obtained automatically by applying Neighbor-Join and BioNJ algorithms to a matrix of pairwise distances estimated using the Tamura-Nei model, and then selecting the topology with superior log likelihood value. The tree with the highest log likelihood (− 1,427,221.59) is shown. Internal colours correspond to clusters K1 to K5 (see panel **a**). **c** PCoA visualization of the genetic relationships between members of the association panel. Coloured symbols in the phylogenetic tree and the PCoA represent accessions from different cultivar group indicated in the legend

Partitioning of the studied germplasm collection based on 37,317 SNPs and independent STRUCTURE imputations at a range of K-values from 1 to 14 and Evanno’s test (Additional file 1: Figure S3) showed K = 5 as the most likely number of subpopulations (Fig. 7a). Two large subgroups were detected including 85 (K = 2) and 114 (K = 3) individuals, whereas the remaining subpopulations were composed by 18 (K = 1), 12 (K = 4), and 2 (K = 5) accessions (Table S4). The remaining 12 genotypes were classified as admixed. All the accessions groups were represented in the detected subpopulations except for K5 which included only two LS genotypes.

The maximum likelihood-based phylogenetic analysis based on Tamura-Nei model divided the collection into two main clusters: a smaller one mostly including LS accessions, while the remaining genotypes were clustered in different subgroups in the second branch. The dendrogram better highlighted the presence of groups based on varietal types (Fig. 7b). The genetic relationships based on PCoA (Fig. 7c) confirmed the presence of specific sub clusters for the established accessions groups, highlighting a major dispersion of the improved cultivars. The degree of relationship between elements of phenotypic and molecular matrices showed significant correlations between morpho-agronomic and genetic data (r = − 0.836, *P* < 0.0001).

The estimate of *r*^*2*^ for all pairs of linked SNP loci were used to assess the extent of linkage disequilibrium decay (Additional File 1: Figure S4). Across the genome, a rapid LD decay was observed (*r*^2^ < 0.20) within less than 50 kb genomic regions.

### Genome-wide association mapping

**Fig. 8:**
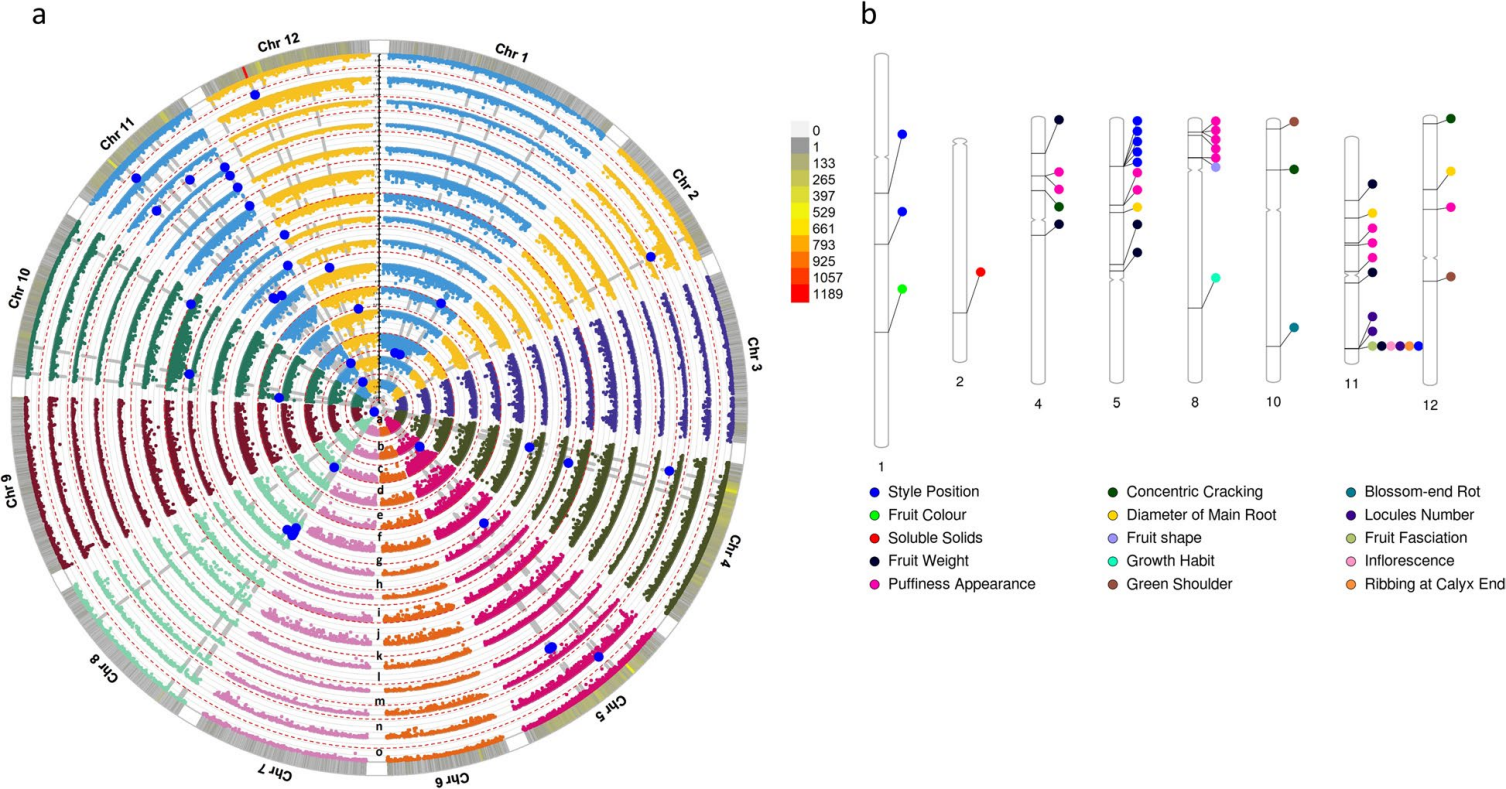
Genome wide association analysis. **a** Circos plot diagram showing associations for 15 traits. The dashed red line indicates significant threshold (−log10 *p*-value). For each chromosome is showed the (SNP) density in the tomato collection. The legend scale indicates the number of SNPs within 1 Mbp window size. Significant peaks are represented by blue dots. Each trait is represented by a concentric circle: a) Growth Habit, b) Inflorescence, c) Style Position, d) Fruit Shape, e) Green Shoulder, f) Fruit Color, g) Puffiness, h) Ribbing Calyx End, i) Concentric Cracking, j) Blossom-end rot, k) Fruit fasciation, l) Locules Number, m) Fruit Weight, n) Soluble Solids, o) Diameter of the Main Root. **b** Significantly associated SNPs, their chromosomal position and cluster of variants on eight tomato chromosomes. Circles represent the association between one genetic variant and one trait. Colors distinguish phenotypes

**Fig. 9:**
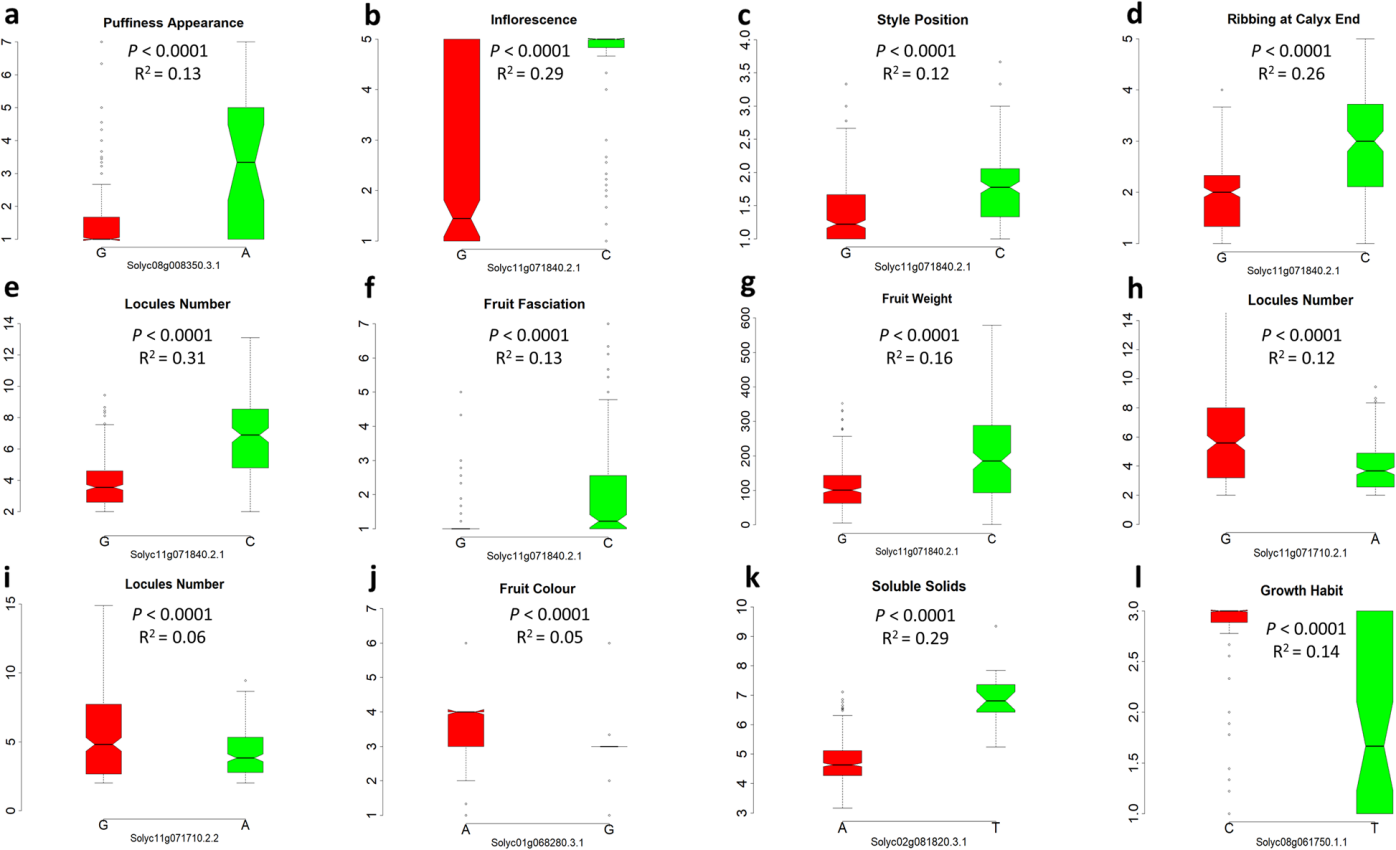
The effect of SNPs falling within gene regions. Notched box-plots showing the phenotypic performances of traits due to associations within genes. Red boxplot indicates the major allele, green boxplot indicates the mutated minor allele

Genome wide association analysis was performed following a univariate composite linear mixed model implementing structure covariates (five sub-populations) as fixed effects and genetic relatedness (kinship matrix) as random effect. For fifteen traits, 47 significantly associated variants on 8 chromosomes, were consistently identified in both environments (Table 3). Twelve additional associations were found for six traits in only one of the sites (Table S5). Most peaks were in intergenic regions at a distance of 2.75 kb to 1.6 Mb from known genes, and associations underlying seven genomic regions were found on five chromosomes. A circular plot summarizing GWA analysis and singular Manhattan and QQ plots are shown in Fig. 8a and Additional File 1: Figure S5. The largest number of associations were found for fruit puffiness appearance (Fig. 8b). A *ribosome maturation factor* located on the top of chromosome 8 was associated to the increase of puffiness (Fig. 9a). The underlying candidate gene was part of a cluster of variants encoding for specific metabolic enzymes and for zinc finger proteins which are involved in several plant growth mechanisms as well as abiotic stress response [21]. In the same region, ~ 6.0 Mbp downstream, an intergenic significant association for fruit shape was detected (Fig. 8b).

**Table 3:** Significant genome-wide associations robustly detected in two environments for morphological, agronomic and root traits using a compressed mixed linear model. For each trait, chromosomal position (in bp), main statistic and effects of each association, corresponding annotated gene or close annotated gene are shown

Trait	Chr^**a**^	Position (kb)	F	-log_**10**_***P*** value	Major/Minor Allele	MAF	Minor Allele Effect	Candidate/Nearby gene	SNP position relative to the candidate gene^**b**^	Candidate gene annotation
Growth Habit	8	47,571,836	23.94	1.31E-06	C/T	0.12	−3.2	Solyc08g061750.1.1	0.0 kb	**Adenylate isopentenyltransferase**
Inflorescence	11	53,260,458	16.85	8.57E-08	G/C	0.56	2.78	Solyc11g071840.2.1	0.0 kb	**SUN-like protein 31**
Style Position	1	34,753,298	13.98	1.27E-06	A/G	0.31	−1.39	Solyc01g034213.1.1	+ 23.60 kb	Unknown protein
	1	47,749,676	14.03	1.20E-06	C/T	0.26	−2.64	Solyc01g056485.2.1	−21.89 kb	sentrin-specific protease 1-like
	5	11,580,510	15.08	4.49E-07	T/C	0.06	0.11	Solyc05g015610.3.1	+ 4.98 kb	Histidine kinase 3
	5	11,580,511	14.63	6.87E-07	G/A	0.06	0.09	Solyc05g015610.3.1	+ 4.98 kb	Histidine kinase 3
	5	11,580,560	15.08	4.49E-07	G/T	0.06	0.11	Solyc05g015610.3.1	+ 5.02 kb	Histidine kinase 3
	5	11,580,572	15.08	4.49E-07	T/A	0.06	0.11	Solyc05g015610.3.1	+ 5.03 kb	Histidine kinase 3
	5	11,580,755	15.08	4.49E-07	A/G	0.06	1.11	Solyc05g015610.3.1	+ 5.22 kb	Histidine kinase 3
	11	53,260,458	17.81	3.51E-08	G/C	0.35	−0.25	Solyc11g071840.2.1	0.0 kb	**SUN-like protein 31**
Fruit shape	8	9,391,514	21.53	1.01E-06	C/T	0.07	2.15	Solyc08g016791.1.1	−0.73 kb	Unknown protein
Green shoulder	10	1,855,401	14.69	6.48E-07	G/A	0.28	−0.27	Solyc10g007720.4.1	+ 0.42 kb	Conserved oligomeric Golgi complex subunit 5
	12	41,393,666	14.22	1.00E-06	G/A	0.10	− 0.86	Solyc12g035620.2.1	−48.38 kb	Polynucleotidyl transferase
Fruit Colour	1	70,208,801	12.36	1.10E-06	A/G	0.01	−0.52	Solyc01g068280.3.1	0.0 kb	**CHROMATIN REMODELING 24**
Puffiness Appearance	4	14,315,417	14.89	5.40E-07	G/T	0.13	0.78	Solyc04g028560.5.1	+ 179.117 kb	Lysine-specific demethylase REF6
	4	14,315,418	14.89	5.40E-07	A/C	0.13	0.77	Solyc04g028560.5.1	+ 179.118 kb	Lysine-specific demethylase REF6
	5	21,484,118	14.27	9.62E-07	A/G	0.06	1.04	Solyc05g160290.1.1	−81.983 kb	Zinc-finger homeodomain protein 3
	5	21,484,211	14.27	9.62E-07	G/A	0.06	1.04	Solyc05g160290.1.1	−81.890 kb	Zinc-finger homeodomain protein 3
	8	2,778,672	38.57	1.14E-09	C/T	0.06	1.19	Solyc08g008340.4.1	+ 2.75 kb	Nucleoside diphosphate kinase
	8	2,783,032	31.66	3.13E-08	G/A	0.06	1.69	Solyc08g008350.3.1	0.0 kb	**ribosome maturation factor**
	8	2,782,603	16.04	1.81E-07	G/A	0.08	0.98	Solyc08g008350.3.1	0.0 kb	**ribosome maturation factor**
	8	3,626,105	27.14	6.83E-12	G/A	0.06	1.36	Solyc08g014080.4.1	−14.64 kb	RING/FYVE/PHD zinc finger superfamily protein
	8	9,290,304	32.58	2.01E-08	C/T	0.00	4.20	Solyc08g016790.1.1	−37.40 kb	Unknown protein
	11	26,261,071	18.47	1.91E-08	T/G	0.11	−5.44	Solyc11g160970.1.1	+ 192.90 kb	Polyprotein
	11	26,877,894	20.87	2.07E-09	A/G	0.10	−6.03	Solyc11g045670.3.1	+ 98.82 kb	S-acyltransferase
	11	33,544,861	23.64	1.62E-10	G/T	0.07	−6.68	Solyc11g042710.3.1	+ 4.51 kb	(+)-neomenthol dehydrogenase-like
	12	23,114,935	43.24	1.28E-10	C/T	0.004	5.79	Solyc12g026360.1.1	−76.66 kb	Unknown protein
Ribbing at Calyx End	11	53,260,458	37.78	6.07E-16	G/C	0.35	0.95	Solyc11g071840.2.1	0.0 kb	**SUN-like protein 31**
Concentric Cracking	4	18,063,194	14.08	1.16E-06	C/A	0.12	−2.54	Solyc04g026150.2.1	−11.09 kb	homeobox-leucine zipper protein ROC8-like
	10	12,292,223	17.98	3.02E-08	G/A	0.08	−3.69	Solyc10g160680.1.1	+ 5.34 kb	GrpE protein homolog
	12	1,284,242	13.95	1.30E-06	A/G	0.07	0.69	Solyc12g006850.2.1	+ 15.65 kb	LELKT1GEN L.esculentum potassium channel
Blossomendrot	10	57,291,816	14.20	1.02E-06	A/G	0.20	−1.67	Solyc10g074680.2.1	+ 19.26 kb	Protein SCARECROW
Fruit fasciation	11	53,260,458	24.33	8.81E-11	G/C	0.35	0.69	Solyc11g071840.2.1	0.0 kb	**SUN-like protein 31**
Locules number	11	53,189,222	13.82	1.30E-06	G/A	0.43	−0.36	Solyc11g071710.2.2	0.0 kb	**Thylakoid lumenal P17.1 protein**
	11	53,189,305	14.71	6.34E-07	G/A	0.49	−0.53	Solyc11g071710.2.1	0.0 kb	**Thylakoid lumenal P17.1 protein**
	11	53,260,458	43.58	4.30E-18	G/C	0.35	3.93	Solyc11g071840.2.1	0.0 kb	**SUN-like protein 31**
Fruit weight	4	8,517,135	15.39	3.37E-07	A/C	0.12	16.29	Solyc04g017860.1.1	−16.52 kb	Aminotransferase-like
	4	29,500,083	13.89	1.28E-06	C/A	0.11	−198.71	Solyc04g024530.3.1	+ 46.73 kb	Actin-related protein
	5	36,583,094	17.72	3.80E-08	A/G	0.06	275.06	Solyc05g025770.1.1	+ 1685.55 kb	asparagine-rich protein-like
	5	38,204,910	17.77	3.61E-08	T/C	0.07	− 183.23	Solyc05g025920.3.1	+ 22.06 kb	Auxin responsive SAUR protein
	11	15,470,616	14.55	7.34E-07	T/C	0.10	−52.13	Solyc11g160550.1.1	−11.30 kb	Peptidyl-prolyl cis-trans isomerase PASTICCINO1
	11	36,433,332	15.86	2.18E-07	A/G	0.11	−114.62	Solyc11g040390.2.1	−14.99 kb	Aspartokinase-homoserine dehydrogenase
	11	53,260,458	32.51	5.93E-14	G/C	0.35	152.71	Solyc11g071840.2.1	0.0 kb	**SUN-like protein 31**
Soluble Solids	2	43,609,921	14.64	6.77E-07	A/T	0.05	1.33	Solyc02g081820.3.1	0.0 kb	**Haloacid dehalogenase-like hydrolase**
Diametre of Main Root	5	23,364,578	23.41	2.07E-10	T/A	0.10	−1.52	Solyc05g018743.1.1	−139.73 kb	Retrovirus-related Pol polyprotein from transposon TNT 1–94
	11	19,987,778	37.05	2.41E-09	G/T	0.06	−1.11	Solyc11g028270.1.1	+ 50.96 kb	Bidirectional sugar transporter SWEET
	12	18,088,359	17.94	3.08E-08	T/G	0.43	−0.76	Solyc12g082795.1.1	+ 19.66 kb	Unknown protein

^a^ Chromosome

^b^ Upstream and downstream SNPs close to candidate genes are specified with “–” and “+,” respectively. 0 indicates that SNPs falls within the candidate gene

Colocalization of associations for several flower and fruit traits including, inflorescence, style position fruit fasciation, number of locules, fruit weight and ribbing at calyx end was found for *sun-like protein* 31 (*Sl*SUN31). In all instances, the minor allele determines an increase of the trait (Fig. 9b-g). In the same genomic region, 71.15 kb upstream, a reduction of locules numbers was found associated with a *thylakoid lumenal protein* (Fig. 9h, i).

An additional cluster in the range 11–23 Mbp on the long arm of chromosome 5 enclosed SNPs associated to style position, puffiness, and diameter of the main root, whereas two SNPs for fruit weight were found associated downstream at 36–38 Mbp. All detected variants fall in intergenic regions closely linked to candidate genes underlying common physiological and developmental processes in plants.

Significant associations within genes were found on the long arms of chromosomes 1, 2 and 8 for fruit colour, soluble solid contents, and growth habit, respectively. For fruit colour, a *chromatin remodeling* protein (*chr*24) was putatively responsible for the decrease of intensity of fruit colour from red to pink (Fig. 9j), whereas a *haloacid dehalogenase-like hydrolase* (*had*) protein was associated to a consistent increase of soluble solids content (Fig. 9k). The determined growth habit (Fig. 9l) was associated to a single genetic variant on chromosome 8 underlying *adenylate isopentenyltransferase*, an enzyme involved in the biosynthetic pathway of cytokinin in higher plants [22].

Associations in six chromosomes were found within the Spanish’ environment for ripening uniformity, foliage density and radial cracking, whereas two variants associated to radicular crown angle and total yield were found on chromosomes 8 and 11 for the Italian growth site. In all cases, SNPs were in intergenic regions in a range of − 12.25 + 45.48 kb from the candidate gene (Table S5). For density of fine roots, two independent associations were found on the chromosomes 1 and 7 in Italy and Spain, respectively. For the latter, a *basic-helix-loop-helix* (*bhlh*) transcription factor was a candidate for the increase of density of fine roots.

## Discussion

### Breeding potentialities for the established cultivated tomato panel

Since its domestication, tomato has undergone extensive changes leading to the development of diverse morphotypes for plant architecture and fruit features. The advent of modern breeding has targeted the increase of yield through disease resistant cultivars able to satisfy the ever-increasing global demand. Dynamic market trends and changes in consumers’ preferences of recent decades have subsequently widened breeding targets to quality-related traits and shelf-life, for which the potentialities are principally held in vintage cultivars such as local varieties and heirlooms [23]. Years of selection across often marginal environments and alien introgression transfer from wild ancestors broadened the genetic and phenotypic diversity of these germplasm resources providing a suitable reservoir of alleles for breeding in unadapted genotypes and/or for low-input cultivation conditions [24].

In recent years, farms converted to organic systems and related consumer’s demand for organic products have both drastically increased. In this scenario, Southern European countries such as Italy, Spain, and France, represent almost 50% of the total organic acreage area of Europe [25]. While the profitability and convenience of organic farming is still a matter of debate [26], there is an undoubted need to develop suitable cultivars for the sector. Objectives for organic breeding include relevant agronomic, morphological and quality traits’ performance under low-input conditions. To that end, we have explored a diversified cultivated tomato collection representative of the diversity enclosed in ancient and modern varieties, in two organic farms of typical Mediterranean environments.

The considerable level of diversity found for the 37 traits analyzed suggests the presence of suitable cultivars keeping outstanding levels of productivity and fruit quality for cultivation and marketing in the organic sector, as well as an interesting source of variation for breeding purposes. As expected, high heritability values coupled with low variation due to E and G × E interaction were observed for several morphological traits. Interestingly, we found strong genotypic control for fruit weight. This trait is one of the most extensively domesticated in tomatoes and is regulated by major genes/QTLs responsible for the high range of variation [27]. The observed high genetic advance (GAM) combined with high heritability suggests an additive gene action on their expression enhancing conditions for selection [28]. On the contrary, the lower levels of heritability coupled with the low GAM for root architecture highlights how the development of the hypogeal part of plants has a more complex control, with high environmental effect on it. The diameter of the main root and radicular crown angle were lesser influenced by the differences between cultivation sites suggesting the stronger genotypic control for the parts of root anatomy ensuring primary function in plant anchorage and nutrient absorption. This was supported by positive correlations with growth habit, ripening earliness and plant vigour found at both sites, which although non-significant, suggest a link among the main root and traits related to plant fitness. Furthermore, the observed correlations suggest that the root apparatus could play a role in ripening earliness and in the accumulation of soluble solids. Although these two traits are not intercorrelated, the level of sucrose is reported to enhance the ethylene receptor genes and their signaling during tomato fruit maturation [29]. The inverse correlation between °Brix and yield-related traits may be likely due to the minor concentration of the total soluble solids in fruits increasing their size and/or in plants increasing their yield. In addition, we found higher values for chemical traits (SS, AC) and for precocity of ripening in all cultivar groups grown during the late spring-end summer cycle (Italy). This trend could be linked to a synergic effect of increased sunlight intensity and temperature [30, 31]. Overall, the multivariate approach of investigation allowed a better understanding of phenotypic performance in low input conditions for the studied tomato collection as well as of genotypic and environmental factors underlying the variation of traits. Such abundant variation made the core collection suitable for investigation of the genetic basis of traits assessed. Therefore, we performed a genome-wide association mapping analysis.

### Association mapping for candidate genes identification

The tomato panel herein reported is part of a larger collection of 288 individuals previously characterized for its genomic diversity and population structure [32]. By removing the highly redundant accessions and segregating lines, we selected a core set of 244 genotypes used for genome-wide association mapping. The analysis with STRUCTURE performed in the present study, identified a likely number of subpopulations and admixed individuals in agreement with the previous results obtained by using ADMIXTURE [32], confirming the absence of any misinterpretation of ddRAD data in diploid species using both the bayesian approach and the maximum likelihood framework [33]. The collection consisted of five groups of accessions selected based on pre-existing phenotyping information (e.g., fruit colour and morphology, tolerance to stresses), geographic origin and market preferences. Both genomic and phenotypic data highlighted a greater variability within the cultivars group than in the rest of groups, likely due to the more intense breeding activity respect to landraces and heirlooms. This observation is supported by Shouten et al. [34] who reported two boosts of diversity in tomato from the second half of the twentieth century aimed at the introgression of disease resistance alleles from wild relatives as well as at the improvement of quality. Genome-wide association analysis through the CLMM model allowed to identify a total of 59 significant signals associated to 21 traits. This method represents an improvement of the linear mixed model in terms of computational and statistical powers being most suitable to minimize false positives due to population structure [10]. We used the Bonferroni correction for multiple testing to controls the family-wise error. Although conservative, this method allows avoiding any excessive claims, being suitable when background LD exists between SNPs, but they are assumed to be independent (low LD) [35].

Analyses allowed to identify associations in previously reported genomic regions as well as new candidates. Growth habit in tomato is regulated by self-pruning, for which, the loss-of-function mutated gene (*sp*) is one of the most common used for breeding modern cultivars [36]. We found a single mutation not previously reported for the candidate gene underlying cytokine biosynthesis, essential hormones for growth and development [22], suggesting a possible role for plant habit.

Fruit shape, weight, colour, and content of soluble solids are among the most studied traits in tomato for which underlying genes and their function have been described [37]. Major genes responsible for the increase of fruit shape are *ovate* and *sun* conferring fruit elongated as well as *fas* and *lc* controlling locule number. The *sun-like protein* 31 (*Sl*SUN31) detected in this study is part of the 34-member *sun-like* gene family controlling fruit elongation and being reported to be expressed also in buds and young flowers [38]. Similarly, a cluster of flowers and fruit traits associated to the same candidate gene (*Solyc11g071840*) have been described [39]. Furthermore, Mata-Nicolás et al. [18] found the same genomic region associated to the increase of locules number and to uniparous inflorescence, whereas Ruggeri et al. [14]. attributed a fruit weight association to the same locus. This gene is located 2 Mbp upstream of *cell size regulator* (*CSR, Solyc11g071940*) reported as a candidate of *fw11.3,* a major QTL controlling fruit weight by increasing cell size and found in a similar region as *fas* [40]. We also found fruit fasciation associated to *Sl*SUN31 confirming previous reports mapping *fas* in the same region of CSR. Furthermore, *Sl*SUN31 is located at approximately 3 Mbp from the region (*Solyc11g064850*) involved in branched inflorescences of tomato-*fin* mutants [41], although the effect found in our study leads to a compound inflorescence compared to what was previously reported [18]. The associations found on chromosome 11 correspond to regions previously reported to be under selection within wild and cultivated tomato germplasm. Razifard et al. [42] reported selective sweeps for locule numbers at 1.879 Kb from *Sl*SUN31. Lin et al. [43] identified improvement sweeps leading to the increasing of fruit mass in correspondence of *fw11.3*. All these findings highlighted how the distal end of the long arm of chromosome 11 carry selective sweeps signals involved in the domestication and improvement of tomato confirming *Sl*SUN31 as a potential candidate gene for improving traits related to flower and fruit morphology.

Two additional associations were detected for fruit weight on chromosomes 4 and 5, the former in the region of *fw4.1* reported as a major increasing fruit weight in tomato [44], whereas the latter is likely a new genomic region although QTLs for fruit weight on chromosome 5 have been reported in a tomato mapping population [45].

A single association was found for fruit shape, for which the variant was in the range of 29 Mbp from *fs8.1* conferring a blocky and slightly elongated shape [46], and at 47 Mbp from *SlOFP16*, a member of the ovate family proteins key regulators of fruit shape [37]. Furthermore, on chromosome 8, several peaks upstream of the association for fruit shape might be responsible for the variation of puffiness. These findings confirm the presence of a broad region on chromosome 8 containing clusters of genes involved in the variation of morphological characteristics of fruits [47]. Moreover, we found associations on different chromosomes for the puffiness, evidencing the presence of different involved genomic regions as observed earlier [48].

Among quality-related traits, the soluble solids content is important for taste and sweetness in tomato. We detected a single variant on chromosome 2 matching with *Solyc02g081820.3.1,* which encodes for an *Haloacid dehalogenase-like hydrolase*. The candidate gene falls 3 Mbp upstream of a previously identified region encoding for an UV *excision repair protein* (*Solyc02g085840.2*) [15] and in the range of 1.0–3.5 Mbp downstream from transporters of glucose (*Solyc02g079220.2*) [49], transporters of amino acids (*Solyc02g070270*) [50], and proteasome degradation protein (*Solyc02g081700*) [19]. Furthermore, the association maps in the same interval of *ssc2.1* and *ssc2.2*, two QTLs found in tomato RIL and MAGIC populations [51] as well as close to *Brix 2.2* described in a mapping population involving cherry tomato [52]. In addition, within the same genomic region, SSC has been found to colocalize with locule number and fruit weight [19, 53]. Although we did not observe such clustering, our results shed light on the presence of multiple linked genes underlying the soluble solids variation in the interval of 40–45 Mbp of tomato chromosome 2.

Another strong association responsible for the reduced intensity of colour was detected on chromosome 1, at ~ 2 Mbp from the previously identified interval underlying fruit colour and *β*-carotene variation [19]. The candidate gene *chromatin remodeling* 24 (*Solyc01g068280.3.1*) falls in the same genomic region of *SlMYB12*, a gene harbouring the *y* mutation responsible for the reduction of accumulation of naringenin chalcone leading to pink-coloured fruits [54]. The most represented fruits in the studied collection were pink- and red-coloured, therefore, the above-mentioned association suggests the presence of possible additive genes underlying this phenotype on the short arm of chromosome 1.

The root apparatus system represents the hidden part of the plant and in tomato fewer efforts are reported in comparison to other crops for the identification of the genetic basis of its architecture. The number of associations were quite low compared to those found in the epigeal part. Interestingly, for the diameter of the main root, we found a peak on chromosome 11 falling at 50 Kb from a *bidirectional sugar transporter sweet*, which is involved in sucrose efflux through the phloem contributing to its migration in plant tissues and as transient storage reserves in the stem and root [55].

Overall, the extent of the phenotypic and genetic variability enclosed in the present diversified collection and exploited in the association mapping approach allowed to narrow down the genomic regions underlying important traits for tomato breeding as well as identify novel ones. By confirming the previously identified SNPs, the association model adopted validates the approach employed for exploiting our mapping panel suggesting the robustness of the genome-wide associations detected for marker assisted selection and breeding under organic conditions.

## Conclusion

In this study, we performed phenotypic characterization and genome wide association analysis in a broad and heterogeneous cultivated tomato panel grown under organic conditions. We found a broad range of variation in the traditional and improved gene-pool, confirming their potentialities as a reservoir of traits to exploit in genetic improvement. The information obtained consolidate those from previous association mapping studies using mixture of sub-varieties of *S. lycopersicum* (e.g., var. *cerasiforme*) or close related wild species (e.g., *S. pimpinellifolium*). We confirmed the strong involvement of different genomic regions for the traits studied, highlighting a major one on chromosome 11 underlying several flower and fruit traits. These results open the perspective for further approaches through functional validation. Furthermore, these variants, if appropriately validated, are a powerful tool for genomic-assisted breeding in tomato, particularly to obtain new cultivars adapted to organic cultivation.

## Methods

### Plant material

The collection used in this study is composed of 244 cultivated tomato (*S. lycopersicum*) accessions retrieved from the Universitat Politècnica de València (UPV, Spain), the Research Centre for Vegetable and Ornamental Crops (CREA, Italy) and the Tomato Genetics Resource Center (TGRC; USA) genebanks. The panel is part of a larger germplasm collection described by Esposito et al. [32] and consists of five accession groups (AG) including 60 landraces for fresh consumption (FC), 58 long shelf-life landraces (LS) known also as ‘da Serbo’, ‘de Penjar’, ‘de Ramellet’, 44 heirloom varieties (HL), 71 elite cultivars (CL) and 11 breeding lines (BL). Landraces mainly represent the Mediterranean area whereas heirlooms and cultivars come from different regions of the world. Detailed information regarding the accessions is reported in Table S4.

### Field trials and Phenotyping

Accessions were grown in two organic certified fields during the spring-summer season of 2019. The first field was in a private farm of the municipality of Alcàsser (province of Valencia, Spain) (39°37′ N, − 0°44′ E). The second was located at the Research Centre for Vegetable and Ornamental Crops (CREA, Monsampolo del Tronto, AP, Italy) (42°53′ N, 13°48′ E) following an agro-ecological management approach based on both conservation tillage and crop diversification strategies as described by Campanelli and Canali (2012) [56]. At both sites, the average temperatures during the growing season were ~ 20–22 °C with peaks of 38 °C and 40 °C in Spain and Italy, respectively.

According to the typical tomato cycles of the two growing areas, plants were sown at the end of February (Spain) and at the beginning of April (Italy) and transplanted to the open field after 6 weeks. A biodegradable plastic mulch film was used to avoid competition with weeds. Plants were grown with at a density of 2.6 plant/m^2^ using a system of double rows in which canes from adjacent rows were tied together forming a triangle-shaped structure. A drip irrigation system was used for water supply whereas fertilization consisted of the application of an organic fertilizer. At both locations, a randomized block design was followed with three blocks and one replicate, consisting of four plants, per block.

All plants in each block were phenotyped for 5 qualitative and 9 pseudo-qualitative traits related to plant architecture and fruit characteristics as well as for 23 agronomic and root traits of which 9 are pseudo-qualitative and 14 are quantitative. All traits were scored at both locations except root weight which was scored only in Italy. Roots were assessed at the end of the cycle (about 150 days after transplant) firstly removing soil and taking care not to damage the root system, then removing the substrate residues by carefully shaking the roots. Details of traits analyzed scale and method of measurement are listed in Table S6. Details of root characterization are described in Additional file 1: Figure S6. All data have been manually reviewed and curated prior to analysis.

### Phenotypic data analyses

The generalized linear model (GLM) was used to analyze pseudo-qualitative and quantitative traits through the *F* test in the ANOVA to detect significant differences. Trait means differences among the AG and within each AG were compared by using Tukey HSD (honest significant difference) test at *P* = 0.05. Broad-sense heritability (*H*^*2*^) was estimated as the proportion of phenotypic variation σ^2^_p_ due to genotypic variation σ^2^_G_:

1 $${H}^2=\frac{\sigma_G^2}{\sigma_P^2}$$

The phenotypic coefficient of variation (PCV) in percentage was calculated as the ratio of the square root for the phenotypic variation (σ_P_) on the mean (μ) for the considered trait:

2 $$\mathrm{PCV}=\left(\frac{\sigma_P\ }{\mu}\right)\times 100$$

whereas the genotypic coefficient of variation (GCV) was estimated as:

3 $$GCV=\left(\frac{\sigma_G\ }{\mu}\right)\times 100$$

The genetic advance as percent of population mean was also derived as:

4 $$GAM=\left(\frac{\sigma_p\times k\times H2}{\mu}\right)\times 100$$

Where k is the selection intensity at 5% (2.06).

A two factorial linear model was used to determine the effects of the genotype, environment, and their interaction on trait performance:

5 $$\mu ij=\mu + Gi+ Ej+\left(G\times E\right) ij+\varepsilon$$

where μ is the grand mean, “G” is the random effect of genotype “i”, “E” is the environmental main effect corresponding to environment “j”, ε represent the error term. Mean square values (MS), were used to estimate the magnitude of the observed effect while the total sum of squares in percentage (TSS%) was calculated dividing the TSS of the effect by the total TSS. Experimental data were statistically elaborated using R. Correlations across the genotypes for phenotypic traits were calculated using the Spearman’s test at *P* < 0.05 after Bonferroni’s correction for multiple comparisons. The correlogram was constructed and visualized using the Corrplot package implemented in R version 3.0.2 (https://github.com/taiyun/corrplot). Principal component analysis (PCA) was carried out to determine which are the most effective descriptors in discriminating among accessions using the computer package XLSTAT 2012.1 and visualizing the similarities among accessions. The relationships between pairs of phenotypic and molecular data matrices were computed by the Mantel test using Pearson’s *r*-value.

### Population structure analysis

The accessions included in the tomato panel were genotyped by Esposito et al. [32] using Double Digest Restriction Associated DNA (ddRAD) Sequencing. A set of 238,644 SNPs were identified in the set of 244 accessions from mapping raw reads to the *Solanum lycopersicum* SL 4.0 pseudomolecule assembly (www.solgenomics.net). Missing genotype calls were imputed using LD k-nearest neighbor imputation (LD-kNNi) implemented in TASSEL.^13^ Markers were filtered using VCFtools (http://vcftools.sourceforge.net/) with a call rate value lower than 95% and with minor allele frequency (MAF) lower than 5%. After filtering for call rate and MAF, a total of 37,317 SNPs was subsequently used for the downstream analysis. The population structure was inferred using a Bayesian model-based approach integrated by neighbor joining phylogenetic analyses. The model-based analysis was performed using Structure v2.3.4 and admixture model with allele frequencies correlated among populations [57]. Runs were done using 20,000 burn-in cycles followed by 10,000 Monte Carlo – Markov Chain (MCMC) iterations, a number of sub-populations (K) ranging between 1 and 14 with five independent runs for each K. The most probable numbers of sub-populations were determined according to Evanno’s method using Structure Harvester (http://taylor0.biology.ucla.edu/structureHarvester/). Once defined the optimal K value, a second Structure run was repeated at the best K value to maximize the accuracy in determining the membership of each accession. The same parameters as above were used, except for the number of burn-in and MCMC iterations (150,000 and 100,000 respectively). Accessions were considered to belong to a specific sub-population if its membership coefficient (qi) was ≥ 0.50, whereas the genotypes with qi lower than 0.5 at each assigned K were considered as admixed.

A phylogenetic tree was drawn using the Maximum Likelihood method and the Tamura-Nei model with 100 bootstraps. Analyses were conducted in MEGA X software (https://www.megasoftware.net/). Principal coordinate analysis (PCoA) was conducted with the Past 3.04 software using Euclidean distance [58].

The *r*^*2*^ statistic was estimated for each pair of SNPs using Plink v1.09 [59] to investigate the linkage disequilibrium (LD) decay in the entire population by using 37,317 SNP markers. Mean *r*^*2*^ was computed into 1 Kb intervals and LD decay curves was fitted using a non-linear model, with distance (Mb) values on the x-axis and *r*^*2*^ values on the y-axis.

### Genome wide association study

Genome-wide association mapping using a compressed mixed linear model (CMLM) with population parameters previously defined (P3D) was performed in TASSEL [13, 60]. To minimize the confounding effects (e.g., population stratification, unequal relatedness among individuals) the model incorporated population structure covariates (Q matrix, according to the best number of 5 sub-populations) and the kinship (K matrix) estimated using the centered identity by state (IBS) for accounting relationships among individuals. The analysis has jointly implemented phenotypic data from the two locations. For traits for which no association was found, the analysis was performed considering each grown site independently. The significance threshold for marker-trait association was determined after Bonferroni multiple test correction with genome-wide α = 0.05. The *P* values was estimated according to the formula:

6 $$Pvalue=\frac{0.05}{no.\kern0.5em of\ markers\ }$$

Considering 37,317 SNPs, the marker was considered significant when the *P* value was less than 5.873 (−log_10_P = 1.339 × 10^− 6^). The phenotypic variation explained by each marker was the *R*^*2*^-value obtained from CLMM model. Circular Plot, Manhattan plot and QQ plots for GWAS results were produced using the R Package CMplot. The chromosomal location of the genome-wide significantly associated SNPs was displayed using PhenoGram (https://ritchielab.org/software/phenogram).

### In silico candidate gene identification

Physical mapping of significantly associated SNPs and functional annotation of the predicted underlying genes were performed using the *Solanum lycopersicum* SL4.0 genome browser (https://solgenomics.net/jbrowse_solgenomics). For associated SNPs mapping within intron regions, we considered the nearest genes located upstream or downstream of the significant markers. Gene models were blasted against Tomato Genome Proteins (ITAG release 4.0) to determine the gene annotation.

### Availability of data and materials

The genomic raw sequence data analysed during the current study are available in the NCBI-SRA (National Center for Biotechnology Information Short Read Archive) repository database under the accession number BioProject ID PRJNA638535. The phenotypic raw datasets used and analysed during the current study available from the corresponding author on reasonable request.

## Supplementary Information

**Additional file 1.**

**Additional file 2.**

**Additional file 3.**

**Additional file 4.**

**Additional file 5.**

**Additional file 6.**

**Additional file 7.**

## Abbreviations

GWAS: Genome-wide association study
MAF: Minimum allele frequency
CLMM: Compression linear mixed model
LMM: Linear mixed model
LD: Linkage disequilibrium
MCMC: Markov Chain Monte Carlo
PCV: Phenotypic coefficient of variance
GCV: Genotypic coefficient of variance
GAM: Genetic advance as a percentage of the mean
LS: Long shelf life
HL: Heirlooms
BL: Breeding lines
PCoA: Principal coordinate analysis
TSS: Total sums of squares
