# Supplementary material for: Genome wide association mapping for agronomic, fruit quality, and root architectural traits in tomato under organic farming conditions
Source: BMC Plant Biol. 2021 Oct 22;21:481. doi: 10.1186/s12870-021-03271-4 (PMC8532347; doi:10.1186/s12870-021-03271-4)
Supplement: Supplementary file 1 — Additional file 1. [file 12870_2021_3271_MOESM1_ESM.pdf]

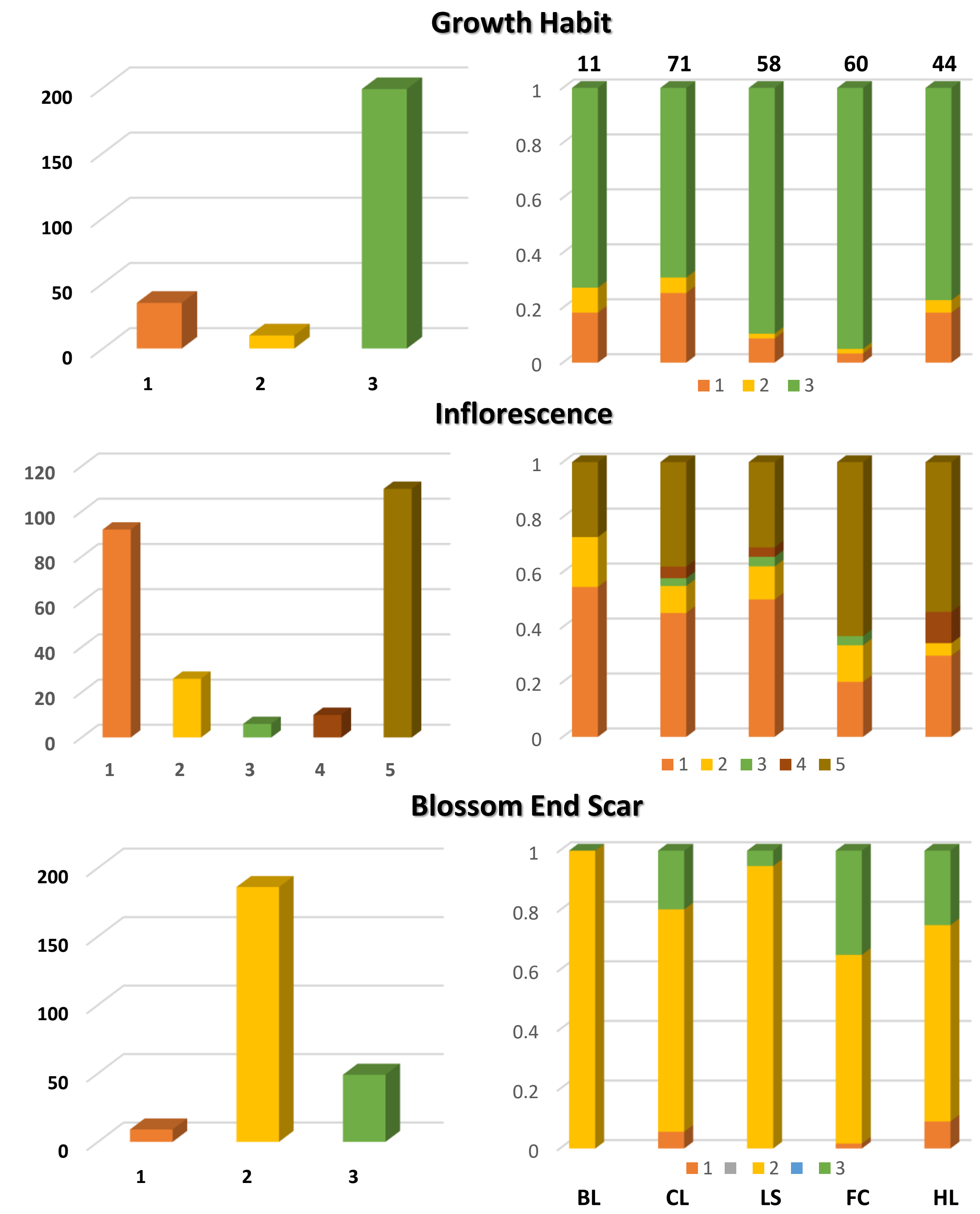

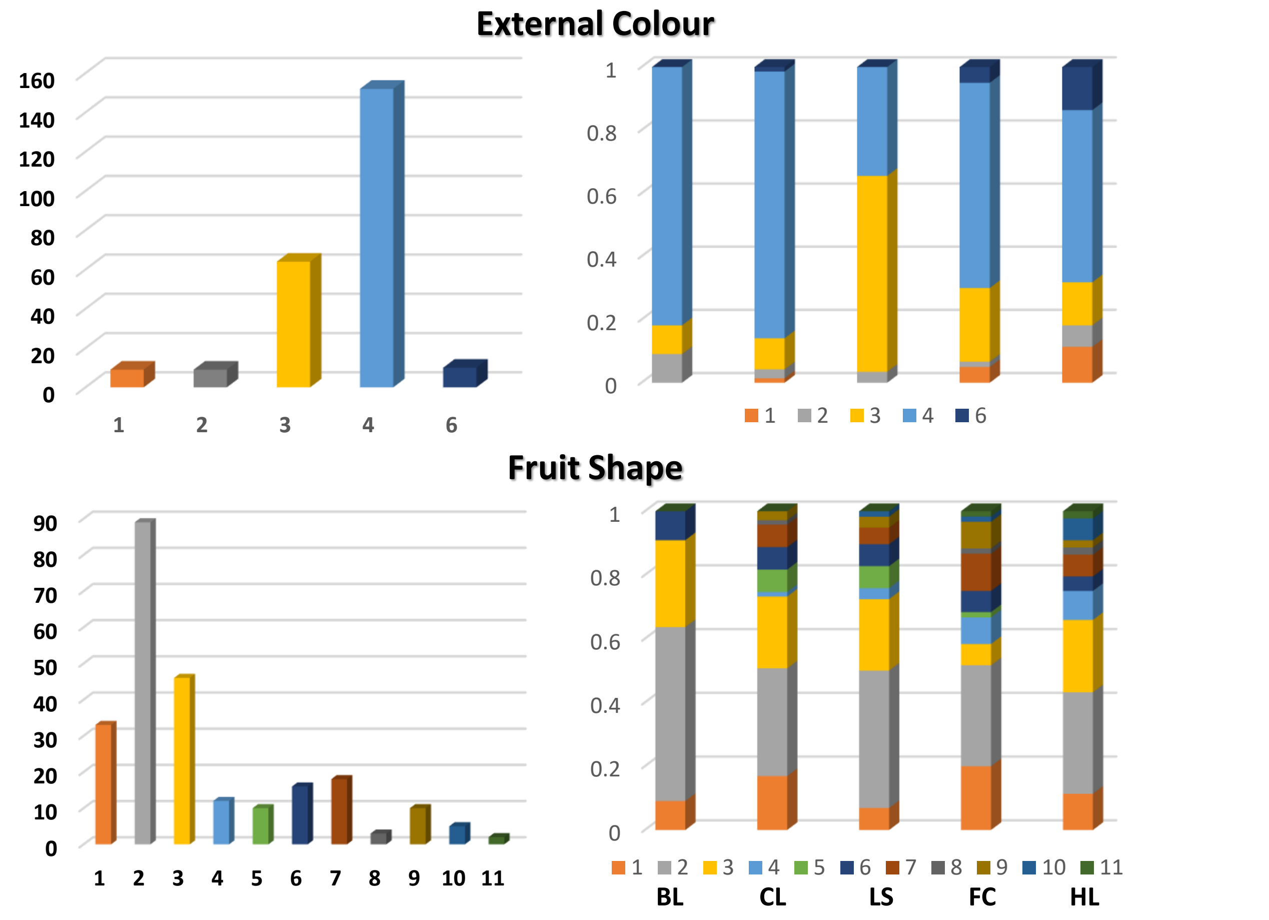

Figure S1: Qualitative traits in the tomato collection. Left: the histograms report the total number of individuals falling in the respective categorical classes for the considered traits. Right: stacked bars indicate the proportion of each class on a total scale of 0-1 for each cultivar group. On the top of each bar are indicated the number of accessions within cultivar groups. Acronyms are indicated at the bottom: BL = breeding lines, CL = elite cultivars, LS = long shelf life landraces, FC = landraces for fresh consumption, HL = heirloom varieties. Details of the measurement scale for each trait are in Supplementary Table 2.

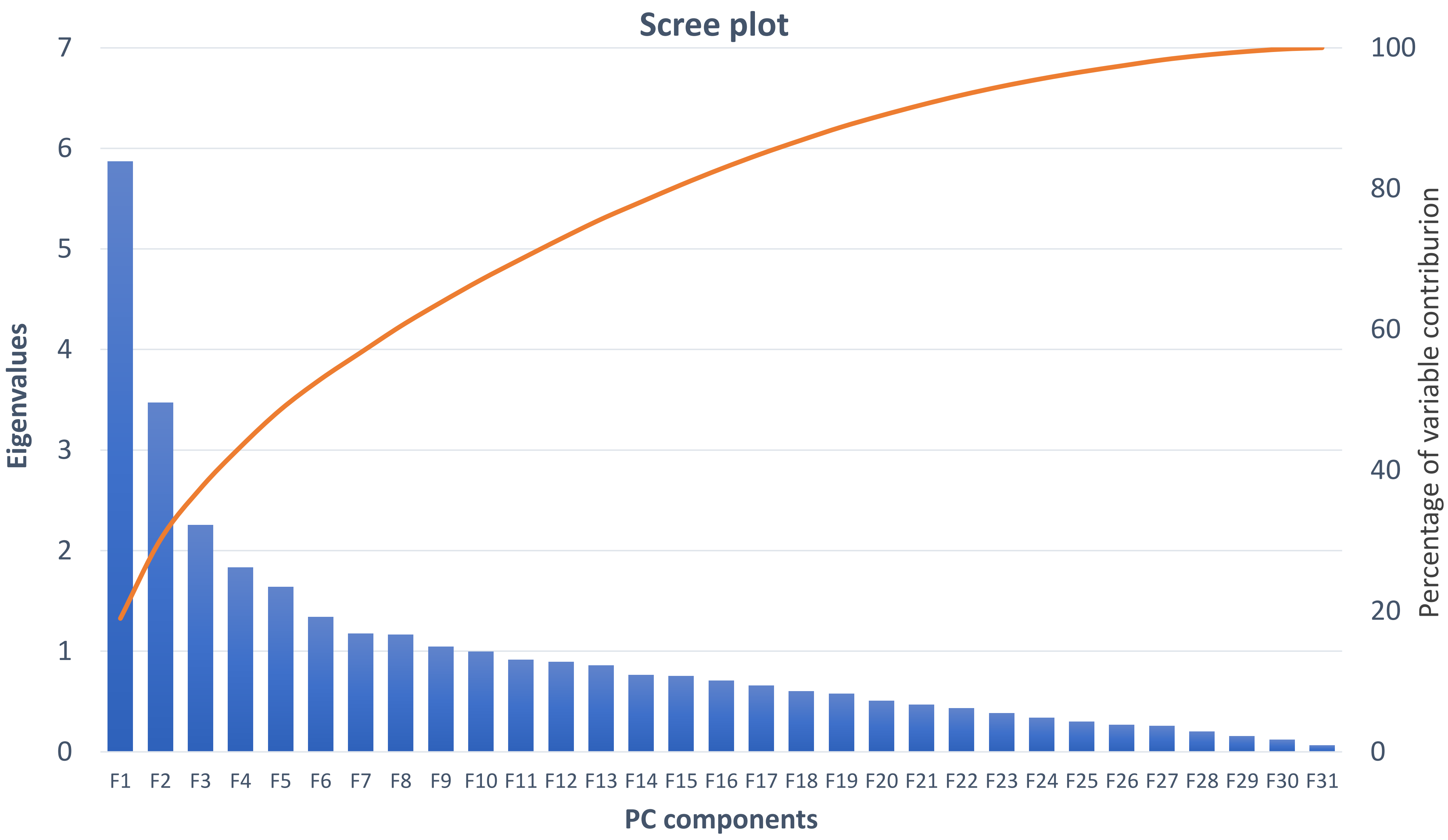

Figure S2: Principal components and related percentage of variation explained. Bars indicates the variation explained by each component. The orange line (above) indicates the cumulative variation of the components from 1 to 31.

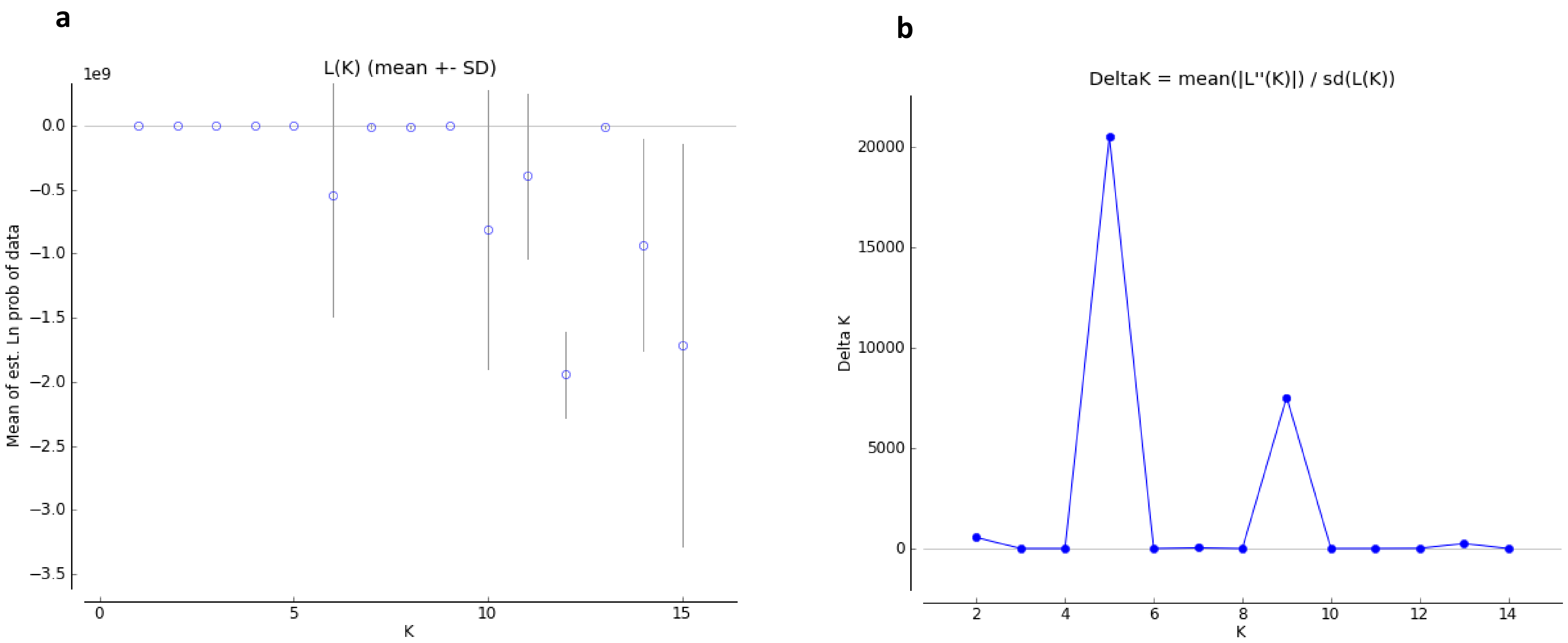

Figure S3: Evaluation of the best grouping number (K) of the Bayesian clustering analysis using the Evanno's method. a) Plot of mean likelihood L(K) and variance for 5 independent runs for each value of K for K = 2–14. b) Evanno's plot generated by STRUCTURE HARVESTER for the detection of the true number of clusters (the most likely value of K). The highest value was at K = 5, indicating that the 244 accessions likely form 5 subpopulations.

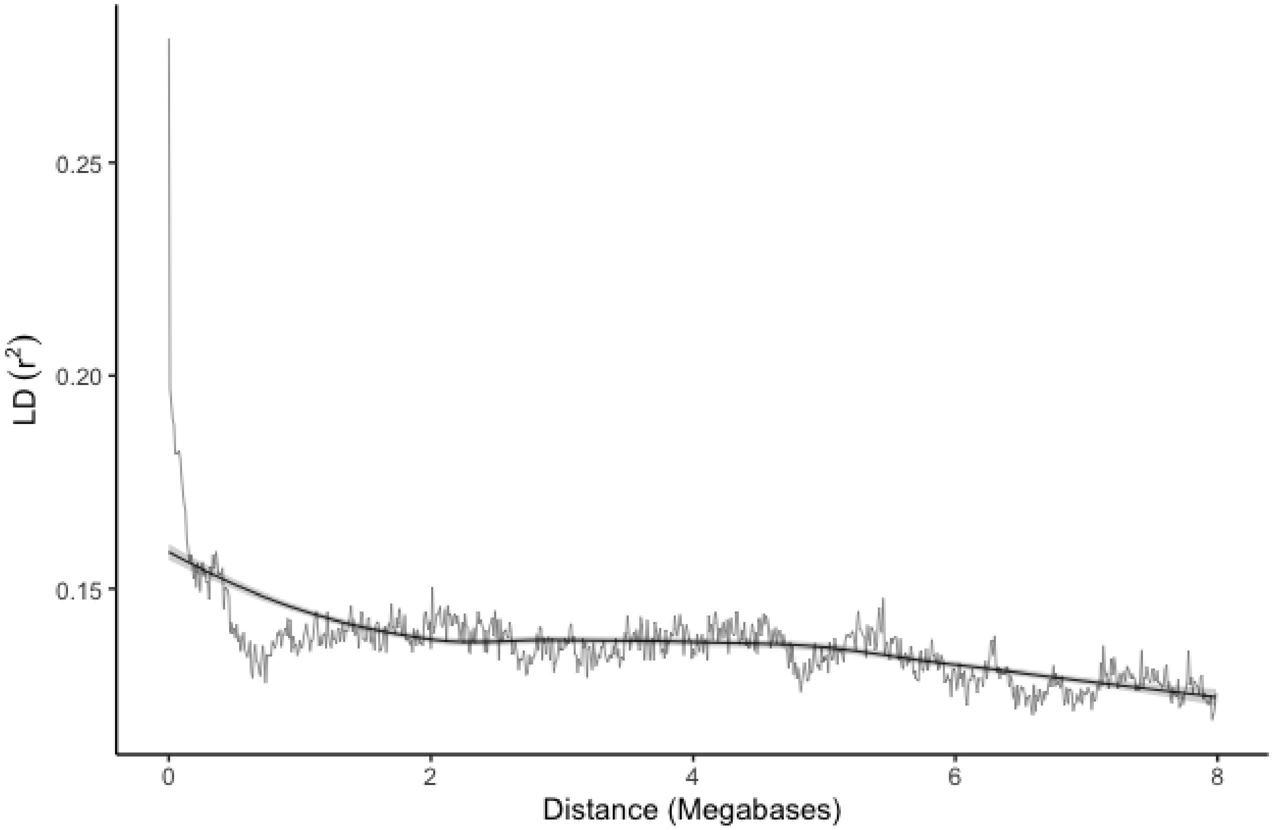

Figure S4: Scatter plot of linkage disequilibrium decay (r2) against the genetic distance for linked SNPs. Solid black curve shows the expected decay of LD in the genome-wide data

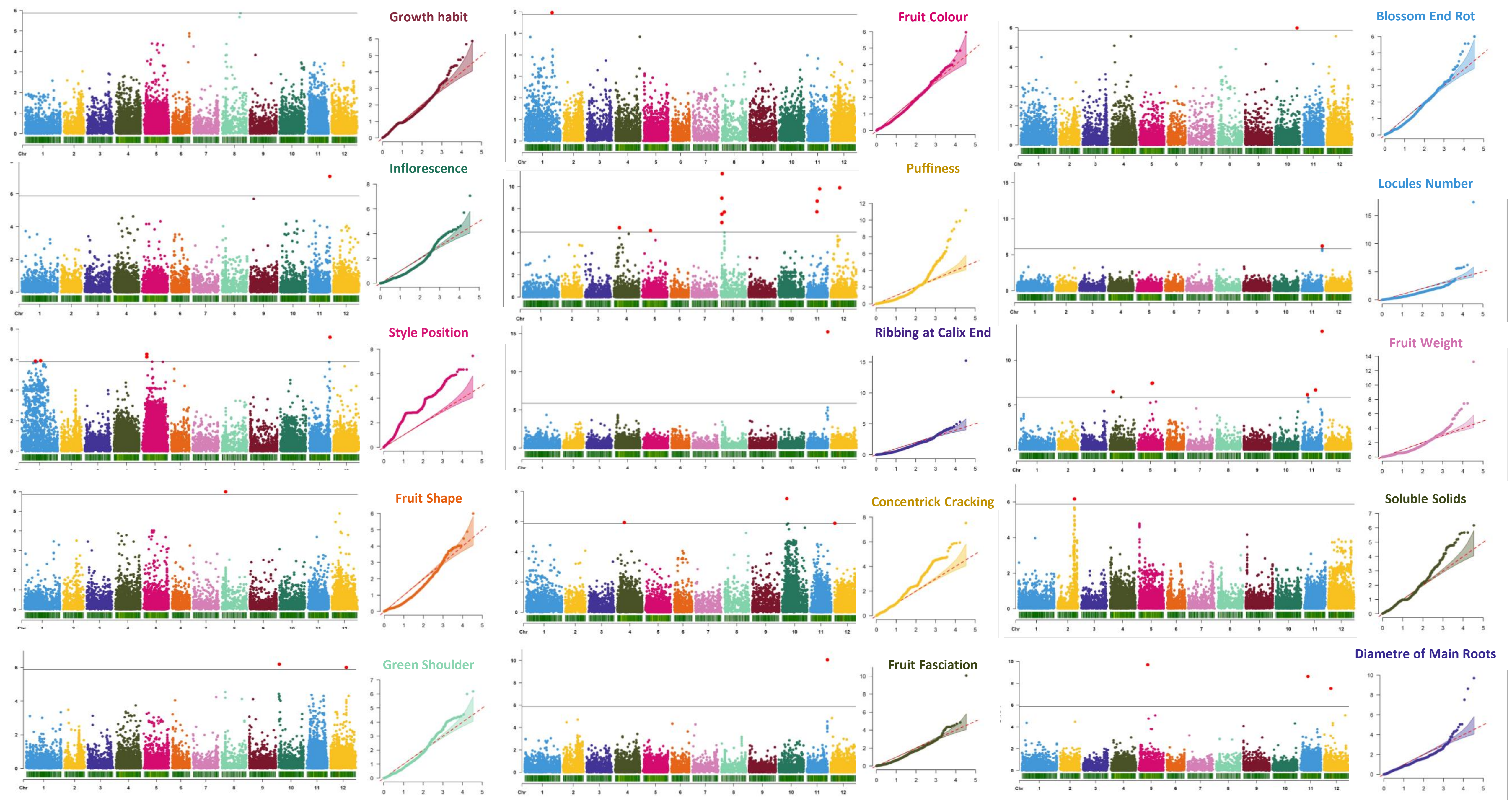

Figure S5: Manhattan and QQ plots for traits associated across two environments. On Y axis of both graphs are reported the $-\log_{10}$ pvalues

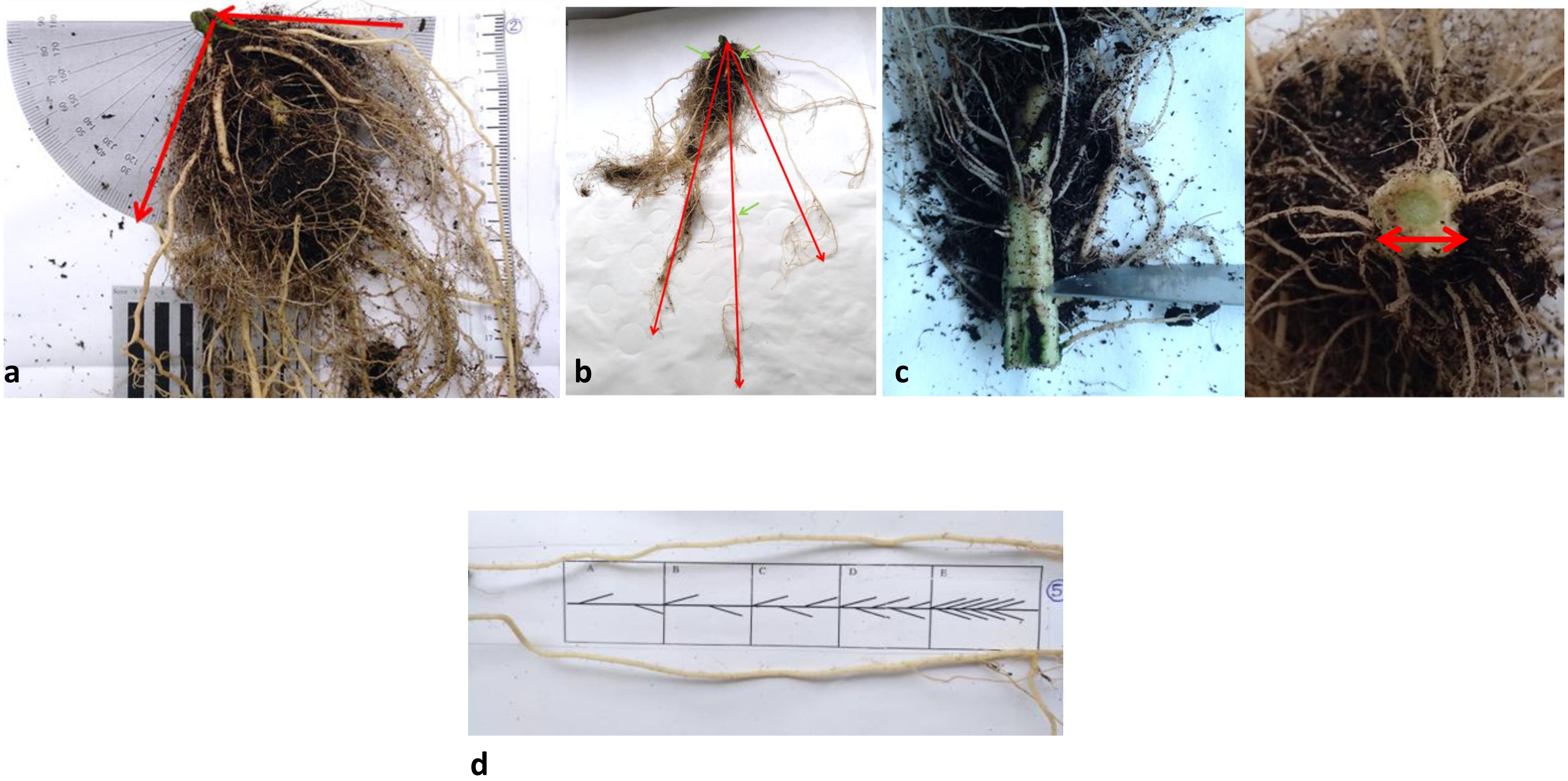

Figure S6: Phenotyping of root apparatus. a) Root crown angle; b) The three longest roots selected for scoring of length and diametre; c) Diametre of the main root at the point of union with the stem; d) Density of fine roots (diametre ≤ 0.5 mm) (a, very scarce/ b, scarce/ c, intermediate/ d, abundant/ e, very abundant).
